# The genetic and proliferation characterization analysis of novel coxsackievirus A12 in Beijing, China

**DOI:** 10.3389/fmicb.2025.1665461

**Published:** 2025-10-08

**Authors:** Zhenzhi Han, Liping Jia, Runan Zhu, Hanhaoyu Fu, Chenbo Lin, Hui Huang, Li Deng, Jianzhao Zhang, Linqing Zhao

**Affiliations:** ^1^Laboratory of Virology, Beijing Key Laboratory of Etiology of Viral Diseases in Children, Capital Center for Children’s Health, Capital Medical University, Capital Institute of Pediatrics, Beijing, China; ^2^Department of Infectious Diseases, Capital Center for Children’s Health, Capital Medical University, Capital Institute of Pediatrics, Beijing, China; ^3^Department of Neurology, Capital Center for Children’s Health, Capital Medical University, Capital Institute of Pediatrics, Beijing, China

**Keywords:** Coxsackievirus A12 (CVA12), mNGs, evolution, proliferation, hand, foot, mouth disease

## Abstract

**Introduction:**

Coxsackievirus A12 (CVA12) is a serotype of Enterovirus A. Its evolutionary and molecular characteristics remain poorly understood.

**Methods:**

The metagenomic Next-Generation Sequencing (mNGS) strategy were used to investigate the viral diversity. The viral isolation, proliferation assays, phylogenetic relationships and recombination events were analyzed.

**Results:**

In this study, nine clinical specimens collected in Beijing, China, during March 2010 to October 2019 were identified as CVA12 positive, among which five were confirmed by mNGS. Then five CVA12 strains were isolated, and the proliferation assays demonstrated the preferential replication of CVA12 in rhabdomyosarcoma (RD) cells, with rapid intracellular replication before being released extracellularly, over Hep-2 cells. Transcriptomic profiling of infected RD cells revealed that the significant up-regulated genes were involved in inflammatory responses and transcriptional regulation (e.g., JUN, FOS), suggesting robust host immune activation. Phylogenetic analysis identified that four strains were clustered into genogroup E, indicating a lineage undergoing active transmission in Beijing, China, the other one into genogroups B. Recombination analysis revealed that strain s7275 exhibited recombination with CVA5 (strain 3,490, GenBank access number OK334538) at the breakpoint position 3,373–6,634, while the others showed recombination with EV-A71 (strain EV71/P1034/2013/China, GenBank access number KP289419) at breakpoint position 3,370–6,645.

**Discussion:**

These findings underscored the genetic diversity and recombination dynamics which provided insights into the evolutionary implications of CVA12, and its proliferation features in RD cells of CVA12. Further research is needed to elucidate the functional mechanisms of CVA12 infection and its role for disease.

## Introduction

The genus enterovirus (EV) belonging to the family *Picornaviridae*, order *Picornavirales*, now contains 15 species assigned to enterovirus A-L, and rhinovirus A-C (Zell et al., 2017). Enterovirus A-D (EV-A, -B, -C, and -D) are species that can infect humans and consist of more than 100 serotypes, including poliovirus, coxsackievirus, echovirus, and some newly identified EVs. Two open reading frames (ORFs) were identified in the genome of EVs, including a typically long ORF (ORF1) of the major region and a second small ORF (ORF2) (Guo et al., 2019; Lulla et al., 2019). A polyprotein ranging from 2,138 to 2,214 amino acids is firstly translated from the ORF1, which then is cleaved into three polyprotein precursors P1, P2, and P3, and further into structural proteins VP4, VP2, VP3, and VP1, non-structural proteins 2A-2C, and non-structural proteins 3A-3D, respectively. In 1999, Oberste et al. discovered that the structural protein-coding region (P1 region) of enteroviruses (EVs) shares consistent genetic information with EV serotype specificity, particularly in the VP1-coding region (Oberste et al., 1999a; Oberste et al., 1999b). The nucleotide sequence variations in *VP1* correlated with neutralization test typing results, making it suitable for molecular typing. The use of VP1 region nucleotide sequence characteristics for EV serotyping gradually replaced the time-consuming and labor-intensive traditional neutralization test method. This approach has been widely recognized as the “gold standard” for EV typing in the molecular era, known as the EV molecular typing method (Brown et al., 1999; Oberste et al., 2002; Zhang et al., 2010; Geoghegan et al., 2015). The prototype strain of CVA12 (Texas-12/AY421768.1) was firstly reported in the United States in 1948, then a few of strains were reported (Oberste et al., 1999b). The phylogenetic analysis of CVA12 based on the full-length *VP1* sequences and whole genome sequences suggested that CVA12 was one of non-EV-A71 and non-CVA16 EVs.

A series of infectious diseases, including acute flaccid paralysis (AFP), hand, foot, and mouth disease (HFMD), myocarditis, aseptic meningitis, and others are associated with EV infections with the development of molecular typing technologies and other methods in recent years (Baggen et al., 2018; Han et al., 2019; Chen et al., 2020). CVA12 has been associated with several clinical diseases, including AFP, and has been frequently detected in cases of HFMD in recent years (Sousa et al., 2020; Hu et al., 2022). The molecular characteristics investigation in Zhejiang province, China illustrated the continual spread of CVA12 in China (Hu et al., 2022). Moreover, CVA12 was detected in HFMD cases reported in Jinan, Shandong Province (Guan et al., 2015). In Thailand, the CVA12 were identified in pediatric patients with HFMD, herpangina and influenza like illness in 2012 (Puenpa et al., 2014). A total of 45 EV serotypes, including the CVA12, were detected in contaminated water in Nigeria in 2021, which represented the first identification of CVA12 in Africa (Majumdar et al., 2021). However, the limitations of current research on CVA12 include limited global surveillance and scarce data on its epidemiology in Beijing (Machado et al., 2024).

In this study, we monitored the epidemiology of EVs in pediatric patients in Beijing, China during March 2010 to October 2019, and nine clinical specimens were identified as CVA12 positive, among which five were confirmed as CVA12 by metagenomic Next-Generation Sequencing (mNGS). Then the proliferations of CVA12 in human rhabdomyosarcoma (RD) or human epidermoid cancer (Hep-2) cells were compared, followed with the virus transcriptomic analysis in RD cells. Moreover, the genomic sequences of CVA12 isolations were amplified using the “primer-walking” strategy and analyzed to reveal the evolutionary dynamics of CVA12.

## Materials and methods

### Sample collection for enterovirus screening and typing

Throat swabs were collected from children who visited Capital Center for Children’s Health, Capital Medical University during March 2010 to October 2019 and diagnosed of HFMD, herpangina, rash and fever illness for EVs screening. The original study was approved by the Ethics Committee of the Capital Institute of Pediatrics (Approval number: SHERLLM2024038). All experimental protocols were approved and the methods were carried out in accordance with the approved guidelines. In the process of EVs screening, viral RNA was extracted from clinical samples using QIAamp Viral RNA Mini Kit (QIAGEN, Hilden, Germany), and real-time reverse transcription polymerase chain reactions (rRT-PCR) for pan-enterovirus (pan-EV), EV-A71, CVA16, CVA6 and CVA10 were performed using the pan EV/EV71/CA16 and the CVA6/CVA10 Nucleic Acid Detection Kit (DaAn, Guangzhou, China) under the reaction condition: 50 °C for 15 min, 95 °C for 15 min, 1 cycle; 94 °C for 15 s, 55 °C for 45 s (fluorescence acquisition), 45 cycles. For samples only positive for pan-EV, complete *VP1* coding region was amplified using the PrimeScript One Step RT-PCR Kit Ver.2 (TaKaRa, Dalian, China) and previously designed primers 486–488 (5’TGGTAICARACIAAITWYGTIGTNCC3’ and 5’GTIGGRTAICCITCITARAACCAYTG3’) and 487–489 (5’ATGTWYGYICCICCIGGIGCNCC3’ and 5’AYIGCICCISWITGYTGNCC3’) to further type EVs, under the reaction cinditions: 50 °C for 30 min, followed by 94 °C for 3 min; 94 °C for 30 s, 42 °C for 30 s and 72 °C for 30 s, 35 cycles (Oberste et al., 1999b; Oberste et al., 2006). The PCR products with expected size were purified using a QIAquick PCR purification kit (Qiagen, Hilden, Germany) for sequencing conducted in SinoGenoMax (Beijing, China) (Hu et al., 2021). Then the sequences of *VP1* coding region were aligned together with the GenBank database using BLAST server.

### Metagenomic next-generation sequencing (mNGS)

For specimens positive for CVA12 identified by *VP1* gene sequencing, metagenomic Next-Generation Sequencing (mNGS) was performed to confirm the exsiting of CVA12. At first, the clinical specimens were homogenized by vortexing, and centrifuged for 10 min at 3,000 × g to get supernatants, which was followed by total RNA extraction using a QIAamp Viral RNA Mini Kit (Qiagen, Hilden, Germany). Then the cDNA of each library was synthesized with SuperScript III Reverse Transcriptase (ThermoFisher, Waltham, MA, USA) and N6 random primers, followed by second-strand synthesis with DNA Polymerase I, Large (Klenow) Fragment (ThermoFisher). Viral sequencing library was prepared following the Illumina TruSeq DNA Preparation Protocol and sequenced on the NovaSeq 6,000 platform (Illumina, San Diego, CA, USA), with the 150 bp pair-end strategy, and carried out by Guangdong Magigene Biotechnology Co., Ltd. (Guangzhou, China).

The outputting data were submitted to the NCBI Sequence Read Archive (SRA). Low-quality bases (PHREAD q < 20) and adaptors were trimmed using Trimmomatic software (version 0.39) (Bolger et al., 2014). Clean reads were aligned firstly to the human reference genome (hg19), and reads matching the human genome were discarded. The remaining reads were *de novo* assembled using Trinity software (version 2.5.1), and taxonomically assigned using Centrifuge (version 1.0.4) for metagenomic classification (Grabherr et al., 2011; Kim et al., 2016). The assembled contigs were taxonomically assigned using the BLASTn algorithm,^1^ with an e-value cut-off of 1 × 10^−5^. To confirm the assembled contigs containing genomes of CVA12, clean reads were mapped to the reference genome of CVA12 (GenBank accession number AY421768.1) using Bowtie2 (version 2.3.4.3) (Langmead and Salzberg, 2012).

### Virus isolation

Specimens positive for CVA12 were inoculated into 12-well plates with a monolayer of RD cells, and then incubated at 37 °C for viral propagation using Dulbecco’s Modified Eagle Medium (DMEM) supplemented with 2% fetal bovine serum (FBS), 1% penicillin–streptomycin, and 2.5% HEPES (the maintenance medium). The cell lines were provided by the WHO Global Poliovirus Specialized Laboratory in the USA and originally purchased from the American Type Culture Collection (Manassas, VA, USA). Infected cell cultures were harvested when 100% cytopathic effect (CPE) was observed. In the absence of CPE, supernatants of cell cultures were collected and re-inoculated into RD cells for three blind passages. To determine the serotype of virus strains, RT-PCR was performed using the primers 486–488 and 487–489 described above, covering the entire VP1 coding region sequence (Oberste et al., 1999b; Oberste et al., 2006). Then the latest strain among isolations was chosen for the proliferation assays, transcriptomics, and recombination analysis. And the titer of the latest strain was determined by tissue culture infectious dose 50% (TCID_50_), in which 50 μL isolation diluted in tenfolds were inoculated into 96-well plates of RD cells, then washed three times in PBS, cultured in 200 μL maintenance medium, and observed for 7 days to record CPEs. The TCID_50_ of the strain was calculated according to the Behrens-Kärber formula.

### The proliferation analysis of CVA12

To construct a calibration curve for viral nucleotides, total nucleic acid (DNA and RNA) was extracted from 140 μL supernatant of the latest strain using the QIAamp MinElute Virus Spin Kit (Qiagen GmbH, Germany), following the manufacturer’s instructions, which were used as templates to synthesize cDNA through a conventional two-step reverse transcription reaction with random primers, following the manufacturer’s instructions (Invitrogen, Eugene, OR, USA). Then the 5’-UTR gene fragment of CVA12 was amplified using primers CVA12-curve-F (5’GCTCAGCAA GATGCTTGGGGGTTGTACCCA3’) and CVA12-curve-R (5’GATTTCCAT GATTTATTTGCACTATTCAG3’), and inserted into the PGEM-T-easy Vector (Promega, Madison, WI, USA). Then the plasmid was linearized using restriction endonuclease SacI, purified using AMPure XP (Beckman Coulter, CA, USA) and transcribed into RNA *in vitro* using RiboMAX™ Large Scale RNA Production System-T7 kit (Promega, Hilden, Germany), followed by purification. The purified RNA was quantified by Qubit RNA BR Assay Kit (Invitrogen, Eugene, OR, USA) and diluted ten-fold (10^−8^–10^−0^ copies) for rRT-PCR using previously reported primers and probes (Cui et al., 2013; Lu et al., 2021), so as to construct the calibration curve for RNA copies quantification.

To compare the proliferation of CVA12 in RD and Hep-2 cell lines, the latest virus isolation of CVA12 with 100 TCID_50_ determined in RD cells were inoculated into RD and Hep-2 cell lines on six-well plates, respectively in the similar culture conditions. The supernatants and cell pellets were harvested at five time points of post-infection (6, 12, 24, 48 and 72 h) and separated by centrifuging for 10 min at 3000 × g, then both were stored in −80 °C, with each cell pellet kept in 500 μL TRIzol reagent (Invitrogen, Eugene, OR, USA). All assays were in 3 biological replicates (independent infections).

To construct the amplifying curve of CVA12 in different cell lines, RNAs were extracted from 140 μL supernatants and 200 μL cell pellets of both cell lines infected by CVA12 using a QIAamp Viral RNA Mini Kit (Qiagen, Hilden, Germany) or TRIzol reagent, respectively, then the rRT-PCR was used to calculate the RNA copies of CVA12 in 3 biological replicates according to the calibration curve constructed. All data were expressed as the mean ± SD of three biological replicates. Statistical significance was determined by ordinary one-way analysis of variance (ANOVA) with multiple comparisons methods using the GraphPad Prism software.

Then 100 μL supernatants harvested from both cell lines infected by CVA12 at five time points of post-infection (6, 12, 24, 48, and 72 h) were inoculated into RD cells on 96-well plates to determine their TCID_50_.

### Transcriptomic analysis of CVA12 in RD cells

According to the amplifying curve of CVA12 in RD cells, CVA12-infected RD cells harvested at 24 h without fully developed CPE characteristics were chosen for RNA-seq and analysis to get the transcriptomic profiles, which were compared with that of mock RD cells. Total RNA was extracted from 200 μL RD cell pellets infected or uninfected with CVA12 stored in TRIzol reagent at different time points of post-infection. When the RNA integrity number (RIN) was greater than 7 determined by Agilent 2,100 bioanalyzer (Agilent Technologies, CA, USA), the first strand of cDNA was synthesized using random primers through reverse transcription, followed by second-strand cDNA synthesis using RNase H and DNA polymerase, which were then sequenced on the Illumina NovaSeq 6,000 platform (Illumina, San Diego, CA, USA) with a 150 bp paired-end strategy. Differential expressed genes (DEGs) were identified using the DESeq2 software, applying a significance threshold of *p* < 0.01 and |log2FC| > 1. To further explore the biological relevance of the identified genes, Gene Ontology (GO) and Kyoto Encyclopedia of Genes and Genomes (KEGG) pathway analyses were conducted (Wu et al., 2021).

### Whole-genome sequencing (WGS) of CVA12

The full-length genome sequences of the isolated CVA12 strains were amplified by RT-PCR using the“primer-walking” strategy, in which specific primers were designed to close the gaps in the sequences (Supplementary Table S1). The 3′ end of the genome was amplified using an oligo-dT primer as reported previously (Yang et al., 2003; Han Z. et al., 2024). The 5′ end of the genome was amplified using the primers 0001S48, and it was obtained following on the manufacturer’s instructions with the 5′-Full RACE Kit (Takara, Shiga, Japan) (Han et al., 2018).

### Phylogenetic analysis and gene recombination analysis

The full-length genomic sequences and deduced amino acid sequences of proteins from the isolated CVA12 strains in this study were aligned with the EV-A prototype sequences downloaded from GenBank using the ClustalW algorithm implemented in MEGA7 (Kumar et al., 2016). A nucleotide identity matrix was generated using BioEdit software. The full-length *VP1* nucleotide sequences of CVA12 of these isolated strains and those downloaded from the GenBank were used to infer evolutionary relationships using MEGA7, in which the Maximum likelihood phylogenetic trees were constructed using the GTR + I + G model with 1,000 bootstrap replicates (Darriba et al., 2012), and the neighbor-joining (NJ) method with 1,000 bootstrap replicates.

For the screening of recombinant events from the genomic sequences of the isolated CVA12 strains, seven methods (RDP, GENECONV, MaxChi, Bootscan, Chimaera, SiScan and 3Seq) in the recombinant detection program (RDP4, v4.46) were used (Martin et al., 2015). Sequences downloaded from GenBank and closest to the genomic sequences of strains isolated in the study in the Maximum likelihood trees were considered as potential parents. And the phylogenetic incongruence between different regions with *p* values less than 0.05 was strong evidence for recombinant events. Recombinant events should be supported by at least three methods within the RDP4. To confirm these putative recombination events, a smaller data set including recombinant and parental sequences for multiple screenings was developed. The SimPlot program (version 3.5.1) was used for similarity plots and bootscanning analysis, with a 200-nucleotide window moving in 20-nucleotide step (Salminen et al., 1995). Recombination breakpoints were inferred according to the distribution of informative sites, which supported two incongruent tree topologies that maximized the chi-square (χ^2^) sum.

## Results

### Detection and typing of CVA12

From March 2010 to October 2019, a total of 7,652 throat swabs were collected. Among these, 2,898 (37.87%, 2898/7,652) were positive for pan-EV by rRT-PCR, including 357 (4.67%, 357/7,652) positive for EV-A71, 515 (6.73%, 515/7,652) positive for CVA16, 1,298 (16.96%, 1,298/7,652) positive for CVA6, 156 (2.04%, 156/7,652) positive for CVA10, and 572 only positive for pan-EV (7.48%, 572/7652) without initially typing results. Subsequent *VP1* coding region sequence analysis using EV-A and EV-B universal primers identified 364 specimens (4.76%, 364/7,652) belonging to the known serotypes, among which nine (0.1%, 9/7,652) were confirmed as CVA12. The nine CVA12-positive samples were collected from pediatric patients aged 1–4 years, including 5 boys and 4 girls. Among these cases, 4 were diagnosed with HFMD, 3 with herpangina, and 2 presented with fever and rash. No severe cases were found.

### Characteristics of the mNGS in clinical samples

**Figure 1:**
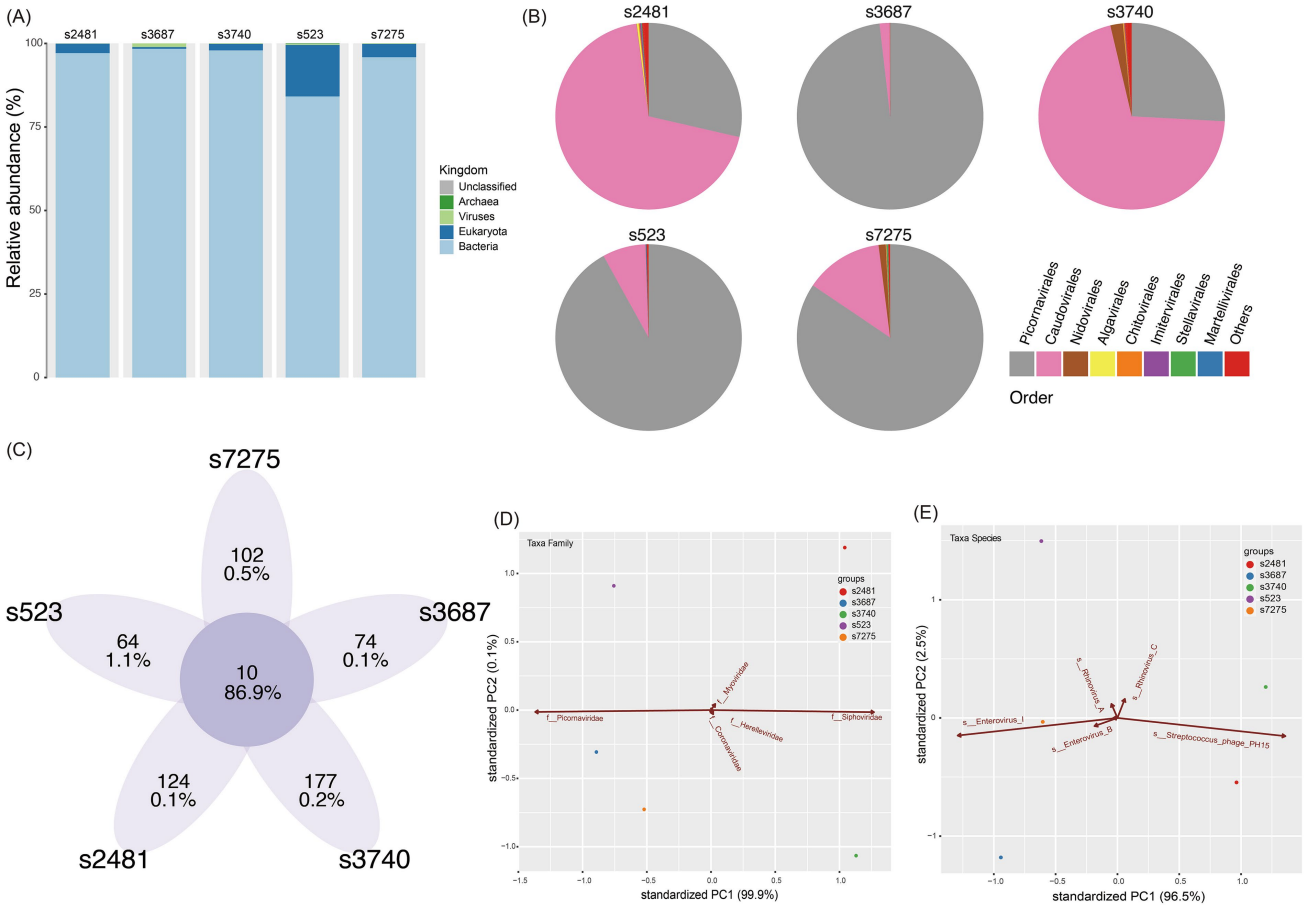
Virome characteristic of five librarie. **(A)** The viral species classification based on the outputting clean data at Kingdom level. **(B)** The pie chart of virome composition at the Order level. The top eight orders of virus are displayed. **(C)** The common and unique taxa of five libraries. The integer is taxa number, while the ratio is the abundancde of viral reads. **(D)** Principal component analysis (PCA) showing the major contributions of different factors (Family) to PC1 and PC2. **(E)** Principal component analysis (PCA) showing the major contributions of different factors (Species) to PC1 and PC2. The first two PCA were used, and the groups are shown in different colors.

**Figure 2:**
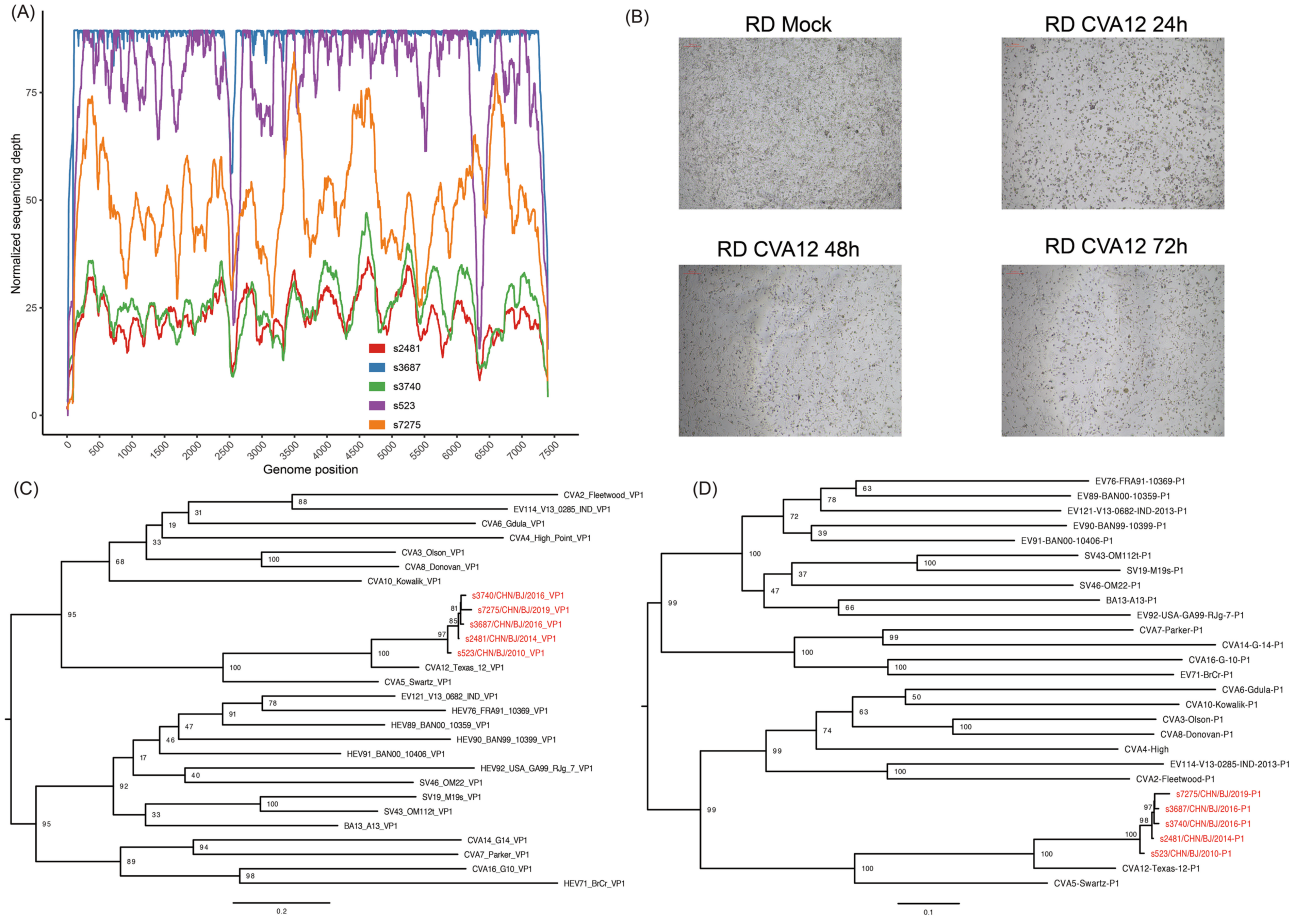
The identification of CVA12. **(A)** The sequencing depth and coverage of all libraries mapping to the reference genome. **(B)** Light micrographs of cytopathic effects of CVA12 strains (strain s7275) at 24 h, 48 h and 72 h post-infection. **(C)** Maximum likelihood tree based on the *VP1* coding region of CVA12 strains in this study and EV-A prototypes. The number at each node represent the bootstra*p* value, and scale bars represent the substitutions per site per year. **(D)** Maximum likelihood tree based on the *P1* coding region of CVA12 strains in this study and EV-A prototypes. The number at each node represent the bootstrap value, and scale bars represent the substitutions per site per year. The red color represent the five strains in this study.

There were five libraries constructed successfully from five clinical specimens of CVA12 with NCBI Sequence Read Archive (SRA) under accession numbers SRR31678006-SRR31678010 (Table 1). And four libraries were not constructed successfully due to low total RNA quantity. In total, 365,428,590 raw reads were obtained from the five libraries, of which 357,803,584 clean reads were identified after quality control. Based on the taxonomical results of outputting reads in each library, the percentage of *homo sapiens* ranged from 0.06 to 7.56% for each library, while 84.1–98.4% were bacteria, and 0.06–1.1% of total reads for each library were viral reads (Figure 1A). The further exploration for viral reads revealed that the order *Picornavirales* dominated the viral distribution for s3687, s523 and s7275 libraries, at 98.2, 91.9 and 84.4%, respectively (Figures 1B,C), and the order *Caudovirales* dominated the library of s2481 and s3740, with 69.3 and 70.5% proportion, respectively, while the order *Picornavirales* was the second one (25.9–28.6%). The principal componenet analysis (PCA) results revealed that family *Picornaviridae* and *Caudovirales* contributed the most to variability (99.9%) in the PCA1, while the PCA2 played a weak role to variation (0.1%) (Figure 1D). Further analysis suggested that species *Enterovirus* of *Picornaviridae* and *Phage PH15* of *Caudovirales* were the most important contributors to variability (96.5%) in the PCA1, while *Rhinoviruses* of *Picornaviridae* were the second most important contributors to variability (2.5%) in the PCA2 (Figure 1E). The CVA12 dominated the major distribution at species *Enterovirus* (15.1–83.9%), which were identified in all libraries (Figures 1B,C, 2A).

**Table 1:** The species information, assembled number of viral sequences and data statistics for each library.

Pool	Number of units	Species	*Homo sapiens (hg)* percentage^a^	Data (reads)^b^	Assembled viral sequences
s7275	1	*Homo sapiens*	2.09%	69,600,152	521
s3687	1	*Homo sapiens*	0.09%	72,110,298	2023
s3740	1	*Homo sapiens*	0.06%	76,639,268	2081
s2481	1	*Homo sapiens*	0.08%	66,511,250	1,373
s523	1	*Homo sapiens*	7.56%	80,567,622	774

a The host percentage was calculated by mapping to the hg38.

b The output data were calculated based on the raw reads of each library.

### Virus isolation and comparison of proliferation features of CVA12 in different cell lines

The nine clinical specimens positive for CVA12 were cultured in RD cells and five strains were harvested with 100% EV-like cytopathic effects (CPE), especially in strain s7275/BJ/CHN/2019 from sample s7275, the latest one (Figure 2B). These strains were confirmed as CVA12 on the basis of their entire *VP1* coding region sequences, which shared 97–99% similarity with known CVA12 strains in GenBank and 80.2–81.9% similarity with the CVA12 prototype strain (Table 2 and Figures 2C,D).

**Table 2:** Pairwise nucleotide and amino acid sequence similarity between the five CVA12 strains and the CVA12 prototype strain (AY421768.1/Texas-12), other prototypes of the EV-A species.

Nucleotide identity (%) [Amino acid identity(%)]										
Region	s7275		s3687		s3740		s2481		s523	
	AY421768.1/Texas-12	Prototype of other EV-As	AY421768.1/Texas-12	Prototype of other EV-As	AY421768.1/Texas-12	Prototype of other EV-As	AY421768.1/Texas-12	Prototype of other EV-As	AY421768.1/Texas-12	Prototype of other EV-As
5’-UTR	80.8	62.5–85.1	82.3	64.2–86.9	82.3	64.4–86.6	82	63.5–86.7	82	63.9–86.5
VP4	75.8	62.8–75.8	76.8	62.8–75.3	76.3	63.2–75.8	76.8	62.8–74.3	76.8	63.2–74.8
	(89.8)	(63.7–88.4)	(89.8)	(65.2–88.4)	(89.8)	(65.2–88.4)	(89.8)	(65.2–88.4)	(89.8)	(65.2–88.4)
VP2	79.8	65.8–72	80.5	65.8–72.5	80.2	66.1–71.8	80.7	66.1–72.1	80.5	65.6–71.2
	(95.2)	(72.6–87.4)	(96)	(72.6–87)	(96)	(72.6–87)	(96)	(72.6–87)	(96)	(72.6–87)
VP3	80.4	64–73.8	80	63.6–73.4	80.2	64.1–73.6	80.6	64.1–73.3	81.5	64.7–73.8
	(95.8)	(70.6–88.7)	(96.2)	(70.6–88.7)	(96.2)	(70.6–88.7)	(96.2)	(70.6–88.7)	(96.2)	(70.6–88.7)
VP1	80.7	55.6–67.9	81.4	55.7–68.1	80.2	55.8–67.1	81.3	56.1–67.5	81.9	55.9–68.2
	(94.5)	(54.9–74)	(95.6)	(54.9–74.4)	(95.6)	(54.9–74.4)	(95.6)	(54.9–74.4)	(95.6)	(54.9–74.4)
2A	77.5	63.3–81.7	79.1	64.2–81.7	77.5	63.7–82	78.4	63.7–81.7	78.4	63.7–82
	(94)	(61.3–96)	(94)	(61.3–96)	(93.3)	(62–95.3)	(94)	(61.3–96)	(94)	(61.3–96)
2B	78.1	56.9–82.4	77.7	56.2–81.4	78.7	56.2–82.4	77.1	56.2–82.4	78.4	56.2–83.8
	(93.9)	(52.5–97.9)	(93.9)	(52.5–97.9)	(93.9)	(52.5–97.9)	(92.9)	(51.5–96.9)	(94.9)	(52.5–98.9)
2C	79.5	63.6–83.4	80.5	63.4–84.5	80.6	63.5–84.7	80.4	63.6–84.1	80.6	63.5–84.2
	(97.5)	(66.2–98.1)	(96.6)	(66.8–97.2)	(97.2)	(67.1–97.5)	(96.9)	(67.1–97.2)	(96.9)	(67.1–97.5)
3A	79	58.2–86.4	76.7	54–84.1	77.1	54.4–84.4	75.9	54.7–84.1	76.3	55.5–85.2
	(97.6)	(58.6–100)	(95.3)	(58.6–97.6)	(95.3)	(58.6–97.6)	(94.1)	(58.6–96.5)	(95.3)	(58.6–97.6)
3B	74.2	48.4–89.3	71.2	46.9–87.8	71.2	46.9–87.8	71.2	46.9–87.8	71.2	48.4–86.3
	(90.9)	(54.5–100)	(90.9)	(54.5–100)	(90.9)	(54.5–100)	(90.9)	(54.5–100)	(90.9)	(54.5–100)
3C	77.7	59.5–84.3	77	56.8–84.8	77	57–84.5	77	56.6–85	76.8	56.4–84.6
	(95)	(59.5–97.8)	(95)	(60.1–98.9)	(95)	(60.1–98.9)	(95)	(60.1–98.9)	(95)	(60.1–98.9)
3D	78	61.5–85.4	78.1	63.5–84.7	77.6	63.7–84.8	77.9	63.7–84.4	77.8	63.5–84.9
	(93.5)	(66.4–96.1)	(92.6)	(67.5–97.6)	(92.8)	(67.5–97.8)	(92.8)	(67.5–97.8)	(93.2)	(67.5–97.8)
3’-UTR	74.7	4.7–89.2	75.2	4.6–87.2	74.1	4.6–86	75.2	4.6–87.2	76.4	4.6–88.3

**Figure 3:**
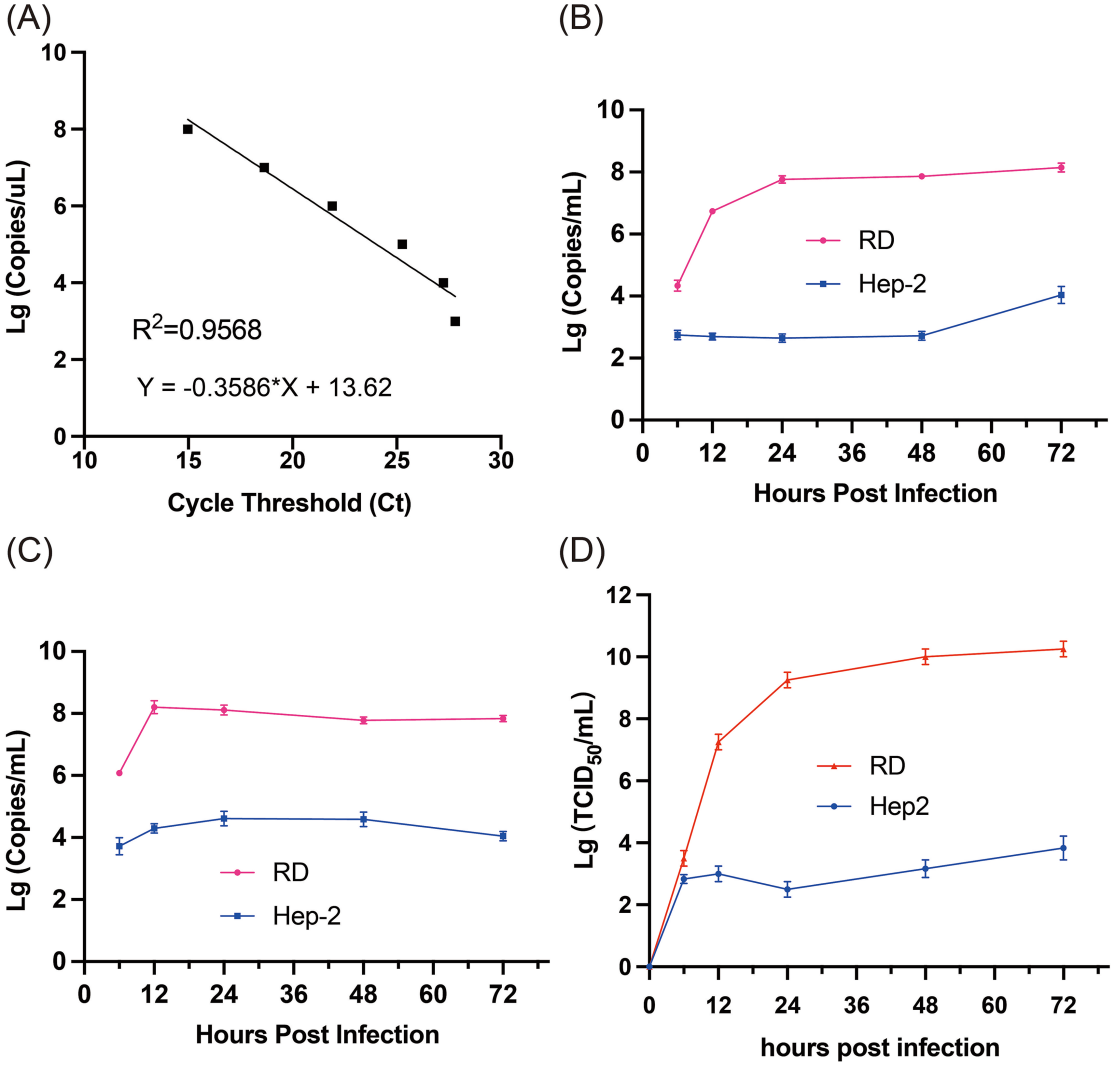
Viral nucleotide loads in growth curve cultures. **(A)** The dynamic range and linearity of the CVA12 real-time assay. The threshold cycle (Ct) values (x-axis) were plotted against tenfold dilutions of CVA12. The parameters of the slope and y-intercept are given by the equation. R^2^, regression coefficient. **(B)** The levels of CVA12 RNA in the culture supernatants at 6, 12, 24, 48 and 72 hpi were determined by qRT-PCR (*n* = 3 biological replicates). The 100 TCID_50_ of CVA12 stock were used to infect RD and Hep-2 cell lines, respectively. **(C)** The levels of CVA12 RNA in the cell lysates at 6, 12, 24, 48 and 72 hpi were determined by qRT-PCR (*n* = 3 biological replicates). The 100 TCID_50_ of CVA12 stock were used to infect RD and Hep-2 cell lines, respectively. **(D)** Virus titer detection in RD and Hep-2 cell lines infected with 100 TCID_50_ of CVA12 stock. The culture were collected at 6, 12, 24, 48 and 72 h post-infection (hpi). All the data are expressed as the mean ± SD of three biological replicates. Statistical significance was determined by ordinary one-way analysis of variance (ANOVA) with multiple comparisons methods using the GraphPad Prism software. *p < 0.05, **p < 0.01, ***p < 0.001, ****p < 0.0001.

The strain (s7275/BJ/CHN/2019) was chosen as template to amplifying its 5’-UTR gene fragment and to construct the standard curve of the rRT-PCR using the Lg (Log 10) value of the RNA copies on the y-axis and the cycle threshold (Ct) value on the x-axis (Figure 3A). The results indicated that the coefficient of determination (R^2^) value of the standard curve was 0.9568, and the minium load of the rRT-PCR detections (LODs) was 1 copies RNA/μL.

Then the strain s7275/BJ/CHN/2019 with 100 TCID_50_ was inoculated into RD and Hep-2 cell lines to evaluate the proliferation features of the strain by rRT-PCR assay. The load of CVA12 RNA increased sharply in the supernatants of RD cells at 6 (10^4.34 ± 0.18^ copies/μl) and 12 hpi (10^6.73 ± 0.01^ copies/μl), and then kept in high level from 24 hpi to 72 hpi, which were in low level in the supernatants of Hep-2 cells until 48 hpi and increased quickly at 72 hpi (10^4.04 ± 0.22^ copies/μl) (Figure 3B). The load of CVA12 RNA in the RD cell pellets reached the plateau at 12 hpi (10^8.21 ± 0.21^ copies/μl), and then slowly decreased following the death of cells (Figure 3C), which reached the plateau at 24 and 48 hpi in the Hep-2 cell pellets, and decreased at 72 hpi (Figure 3C).

The viral titers indicated by TCID_50_ in the RD supernatants (10^7.25 ± 0.204^ TCID_50_/mL) were higher significantly than that of Hep-2 supernatants (10^3.00 ± 0.2^ TCID_50_/mL) from 12 hpi, which reached the plateau at 24 hpi in both cell lines (*p* < 0.0001) (Figure 3D).

### Transcriptomic profiles of CVA12-infected and mock RD cell

**Figure 4:**
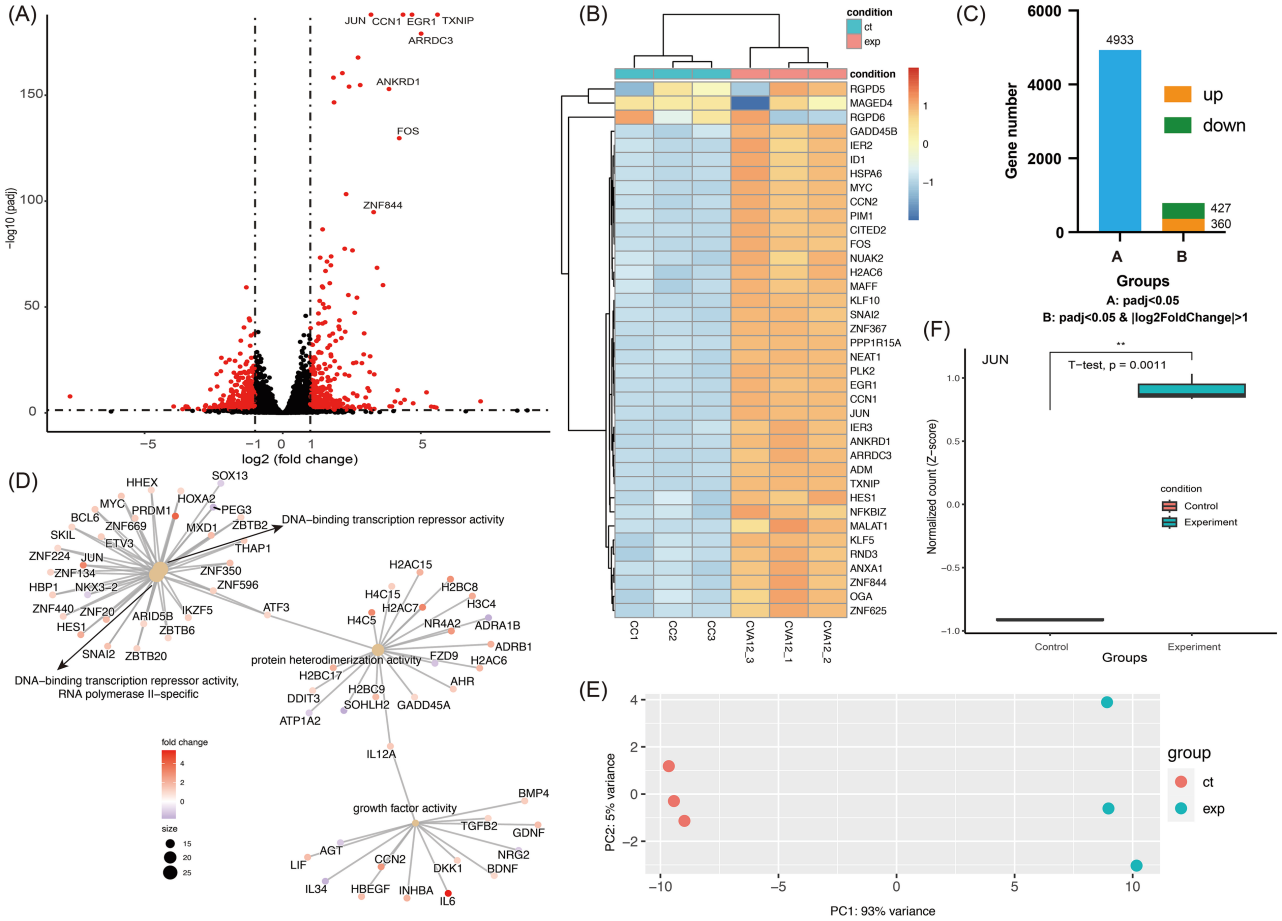
Transcriptomic analysis of CVA12 infected RD cell lines. The 100 TCID_50_ of CVA12 stock were used to infect RD cell lines and the culture were collected at 24 h post-infection (hpi). **(A)** Volcano plot show the differentially regulated genes of CVA12-infected and mock RD cells. The vertical axis represent the negative logarithm of p value (−Log_10_ P) and the horizontal axis represents the logarithm of fold change with base 2 (log2(fold change)). The red points represent the differentially expressed mRNAs with statistical significance (*p* < 0.05 and |log2FoldChange| > 1). The mainly upregulated genes were labeled with gene symbols. **(B)** Heatmap showing the top 40 upregulated genes enriched in regulation between the CVA12-infected and mock RD cells. **(C)** Barplot showing the differentially expressed gene number. Group A represent the gene number with adjusted *p* value < 0.05, and Group B represent the gene number with adjusted p value < 0.05 as well as |log2FoldChange| > 1. **(D)** The cnetplot at the molecular function level details the genes associated with one or more Gene Ontology (GO) terms with adjusted *p* value < 0.05. The color represent the fold changes and the top 4 significant GO terms were given. **(E)** Principal component analysis (PCA) showing the enrichment of mRNAs to PC1 and PC2. The first two PCA were used, and the CVA12-infected and mock RD groups are shown in different colors. **(F)** The normalized count of JUN mRNA at the CVA12-infected and mock RD groups. Statistical significance was determined by Student’s *t*-tests. *p < 0.05, **p < 0.01, ***p < 0.001, ****p < 0.0001.

The transcriptomic profiles of s7275/BJ/CHN/2019-infected RD cells at 24 h of post-infections without fully developed CPE characteristics showed that there were 360 up-regulated and 427 down-regulated genes with *p* < 0.05 and |log2FoldChange| > 1, and 4,933 genes with *p* < 0.05, compared with that of mock RD cells in DEGs analysis (Figures 4A,C). The top 40 DEGs showed a significant up-regulated tendency in CVA12-infected cells (Figures 4B,E). In the CVA12-infected RD cells, amounts of genes related to transcriptional regulation activity and inflammatory response, including JUN, FOS, CCN1, and ZNF844, were up-regulated. And the representative genes expressed abundantly in RIG-I/MDA5, IFN response, and apoptosis pathways were extracted (Supplementary Figure S3). The results revealed that MAVS, IRF7, BCL2 and TP53 showed statistical difference between CVA12 infected and mock groups. GO analysis of the DEGs revealed that the top 5 up-regulated genes were mainly enriched in the positive regulation of DNA-binding transcription repressor activity, growth factor activity, regulation of leukocyte differentiation and regulating defense response (Figure 4D). Especially, the JUN gene, a member of DNA-binding transcription repressor activity, increased significantly (*p* = 0.0011) (Figure 4F). The FOS gene, a member of MAPK/ERK signaling pathway and involveing the inflammatory response, increased significantly (Figure 4B).

### Whole-genome sequences of CVA12

The whole-genome sequences of the five CV-A12 strains were obtained using the “primer walking” strategy, and deposited in the GenBank database with the accession numbers PQ726936-PQ726940. They were 7,384 to 7,399 nucleotides in length with a single ORF of 6,573 nucleotides encoding a single polypeptide of 2,191amino acids. The genome were flanked by a 5′-untranslated region (UTR) of 727–742 nucleotides and a non-coding 3′-UTR of 84 nucleotides. The overall base composition of the five sequences was 27.41–27.64% A, 23.40–24.12% C, 24.16–24.48% G, and 24.12–24.69% T. The nucleotide and deduced amino acid sequences of the five strains showed 90.2–98.4% and 97.7–99.7% similarity with each other, and 79.2–79.7% and 94.7–95% similarity with the CVA12 prototype strain, respectively (Table 2). Higher similarity in the *P1* coding region was shown with the CVA12 prototype strain, whereas higher similarity in the *P2* and *P3* coding region was shown with some other enterovirus prototypes other than CVA12 prototype strain (Table 2). For example, the *P2* and *P3* coding region of the s7275 strain showed 85.4% nucleotide similarity and 95.8% amino acid similarity with the CVA4 prototype strain, respectively, which implied potential recombination events.

### Phylogenetic analysis of CVA12 strains

**Figure 5:**
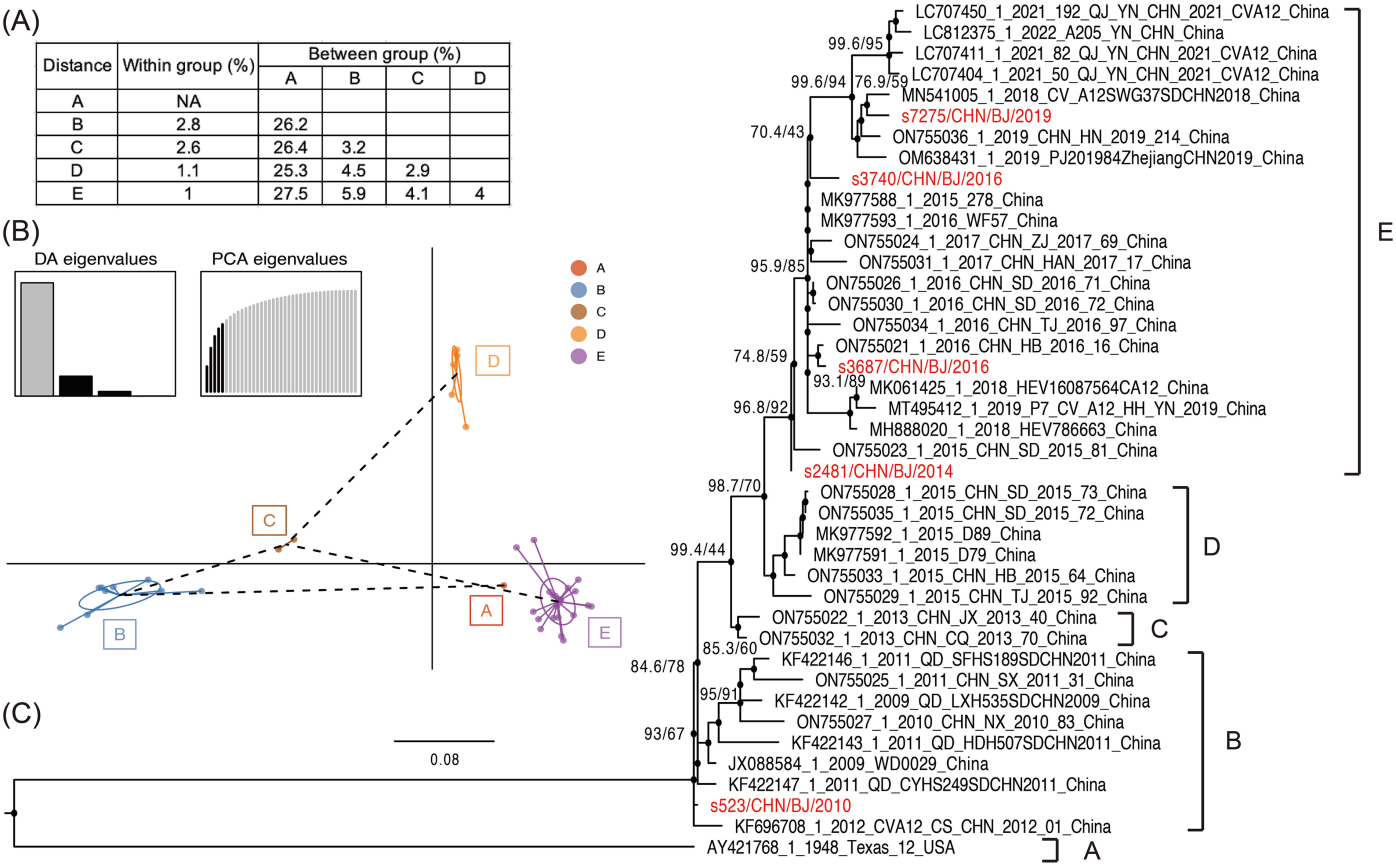
The phylogenetic characteristics of CVA12. **(A)** Nucleotide distances between the different groups and within each group. **(B)** Principal components of CVA12 sequences with genogroups used as prior groups based on *VP1* coding region. Different colors represent prior genogroups, and individual sequences are marked as dots. Results of eigenvalues analysis (PCA and DA) displayed in the inset. Axes represent the frst two principles. PCA, principal components analysis; DA, discriminant analysis. **(C)** Maximum likelihood phylogenetic tree based on the full-length *VP1* coding region, with 1,000 bootstraps and SH-like approximate likelihood ratio tests (SH-aLRT) replicates at each node. The scale bar indicates nucleotide substitutions per site per year. The red colors represent the five CVA12 strains in this study.

A maximum likelihood phylogenetic tree was constructed using the full-length *VP1* coding region of CVA12 sequences available in GenBank, which included the *VP1* coding region of PQ726936-PQ726940 (Figure 5 and Supplementary Figure S2). In the tree, the CVA12 prototype strain (Texas-12) isolated in the USA in 1948 formed a single cluster and designated as genogroup A, presenting 25.3–27.5% genetic distance with other genogroups (Figure 5A). Eight strains isolated from 2009 to 2012 in China clustered together were designated as genogroup B. The s523 strain isolated in 2010 in this study was clustered within the genogroup B. Two strains isolated in 2013 and six strains isolated in 2015, which were identified in China, were designated as genogroup C and D, respectively. Four strains isolated during 2014–2019 in this study and distributed in several lineages were clustered together with nineteen strains isolated from 2014 to 2022 in China into genogroup E (Figures 5B,C). The genogroup E showed 4.0–27.5% genetic distance, comparing with other genogroups (Figure 5A).

### Recombination analysis of the five CVA12 strains

**Figure 6:**
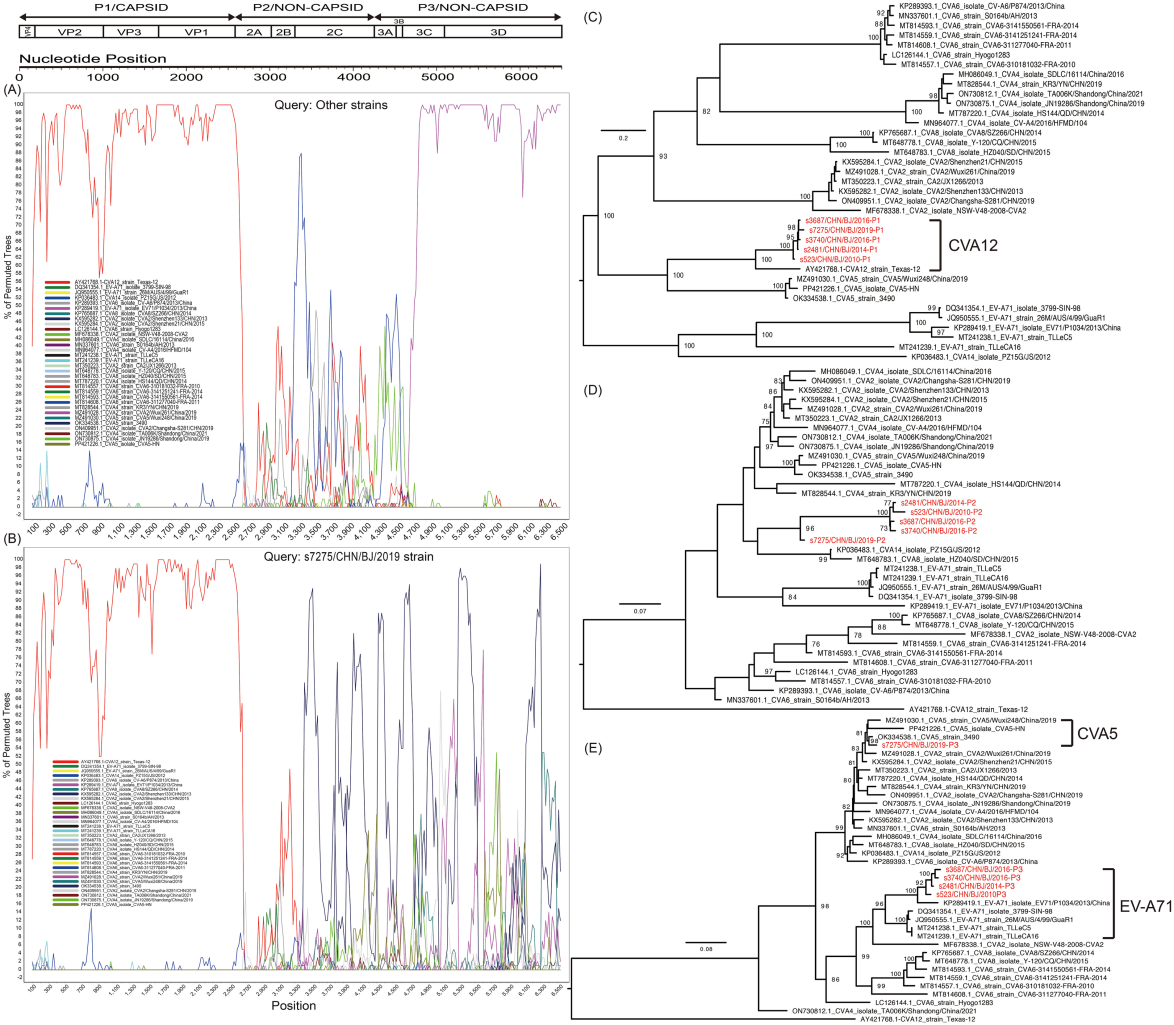
Recombination analysis of CVA12 strains with potential parents, based on the sequence of the open reading fragment (ORF). The s7275 strain and other strains (s3687, s3740, s2481 and s523 together) were used as query sequences. The indications of different colours are shown in the inset. **(A)** Bootscanning plot of other strains; **(B)** Bootscanning plot of s7275 strain. Maximum likelihood trees based on the *P1* **(C)**, *P2* **(D),** and *P3* **(E)** coding region. The sequences are identical to those of **(A,B)**. Numbers at the nodes show bootstrap support values, with 1,000 bootstrap replicates. Scale bars represent the substitutions per site per year. The red colors represent the five CVA12 strains in this study.

Based on the P1 region coding of capsid protein VP1-4 in the maximum likelihood trees, the five strains isolated in the study were clustered together and were closest to GenBank sequence AY421768-CVA12 (Figure 6). Based on the P2 region coding of noncapsid protein 2A-2C, the five strains were clustered together, but with s7275/BJ/CHN/2019 aparted from others, and were closest to GenBank sequence KP036483-CVA14. Based on the P3 region coding of noncapsid protein 3A-3D, the five strains were clustered in two groups, with s7275/BJ/CHN/2019 closest to OK334538-CVA5, and other 4 strains closest to KP289419-EVA71. Therefore, there were two different recombination patterns among the five CVA12 strains. And AY421768-CVA12, OK334538-CVA5, and KP289419-EVA71 were chosen as parents in recombination analysis. The bootscanning analysis revealed that the breakpoints of s7275/BJ/CHN/2019 was at 3373-6634 nt of OK334538-CVA5 in the ORF alignment, which was supported by seven methods of RDP4 (Figures 6A,B and Supplemenatry Figure S4). And the breakpoints of the other four CVA12 strains were at 3370-6645 nt of KP289419-EVA71 and 3,285-3566 nt of MT814608-CVA6.

## Discussion

CVA12, a serotype of EV-A, has been associated with many diseases, especially the HFMD, although has been reported infrequently around the world (Sousa et al., 2020; Hu et al., 2022). Molecular detection and phylogenetic analysis revealed its evolution in locations such as Qingdao and Jinan, China, and its ongoing spread in Zhejiang Province (Liu et al., 2014; Hu et al., 2022). CVA12 was also found in HFMD, herpangina, and influenza-like illnesses in Thailand and in contaminated water in Nigeria, revealing its potential diffusion worldwide (Puenpa et al., 2014; Majumdar et al., 2021). In this study, there were only nine specimens identified as CVA12 among 7,652 throat swabs collected from children during March 2010 to October 2019.

Among the five libraries constructed for mNGS, bacteria were the dominant member, which was consistented with previous studies (Reyes et al., 2012; Liang and Bushman, 2021). The taxonomic analysis highlighted *Picornavirales* as the dominant viral order in three out of five libraries, with *Caudovirales* showing prominence in the other two. These findings suggest that different virus compositions may be related to different clinical specimens collected. The virus taxonomic group shared in all libraries showed that these five samples have common virus components, while the existence of unique low-abundance taxonomic group showed a complex virus ecosystem, which is worth further exploration.

The obvious EV-like CPEs were observed in RD cell lines from five throat swabs. In general, EV-A and some serotypes of EV-B species are more efficiently isolated in RD cells, while EV-C species are more promptly isolated in Hep-2 cells (Bingjun et al., 2008; Bessaud et al., 2012; Sousa et al., 2020). The CVA12 strain proliferated more effectively in RD cells than in Hep-2 cells. The proliferation of CVA12 reached the plateau at 24 hpi in supernatants of RD cells, which presented a similar propagation rate compared with EV-A71 and E30 strains (Guo et al., 2019; Li J. et al., 2022), but at 72 hpi in supernatants of Hep-2 cells. The CVA12 RNA reached the plateau at 12 hpi in the RD intracellular stock, earlier than that in supernatants, indicating that the CVA12 preferentially undergoes rapid intracellular replication before being released extracellularly.

To further investigate the characteristics of CVA12, transcriptomic profiling of CVA12-infected RD cells revealed significant changes in gene expression. The up-regulation of genes associated with transcriptional regulation and inflammatory response highlights a defense against viral infection. GO analysis further confirmed the enrichment of pathways related to leukocyte differentiation and growth factor activity, which are crucial for orchestrating an effective immune response (Li D. et al., 2022; Li J. et al., 2022; Song et al., 2023).

On the basis of the whole-genome sequences of the five CV-A12 strains, these five strains in this study belonged to genogroup B and E, respectively, among five genogroups (A–E) identified. The CVA12 strains circulating in recent years are part of an evolving lineage that shares ancestry with earlier strains identified globally. The placement of four strains within different lineages of genogroup E suggests that these strains are likely descendants of an ongoing transmission chain primarily observed in China (Guo et al., 2022; Hu et al., 2022). Although the s523 strain clustered within genogroup B was rarely detected, its persistent circulation was shown in Beijing, China.

Recombination in enteroviruses is a well-documented mechanism that contributes to their genetic diversity and adaptation (Lukashev, 2005; Kyriakopoulou et al., 2015). The distinct recombination patterns were observed in strain s7275 and the other four strains. The former showed the recombination events with CVA5 strain in the *P3* coding region, comparing with the other four strains that showed recombination with EV-A71 strain in the *P3* coding region. It is widely accepted that two different enteroviruses can only exchange genetic materials in case of coinfection in the same host cell. Due to geographic factors, enteroviruses circulated in populations in different locations that were possibly infected by different dominating enteroviruses, resulting in different opportunities of gene exchange (Kyriakopoulou et al., 2015). The CVA12 strains underwent different recombination processes in different time period, although they were detected in Beijing, China, which presents a similar insight into the recombination potentials (Kyriakopoulou et al., 2015; Han et al., 2018; Guo et al., 2022; Han Z. Z. et al., 2024). The results revealed that the dominant EVs in different time period of the populations, despite being in the same geographical region, were different.

This study reveals critical insights into CVA12’s potential pathogenesis and transmission through its genetic diversity and host interactions. Phylogenetic analysis identified two circulating genogroups (B and E) in Beijing, with genogroup E representing an active transmission lineage, suggesting ongoing viral transmission and adaptation. Recombination events with CVA5 and EV-A71 in the *P3* coding region demonstrate CVA12’s genetic dynamics, which may facilitate immune evasion or expanded tropism. Transcriptomic profiling of infected RD cells showed significant upregulation of inflammatory (JUN, FOS) and immune regulatory genes, indicating robust host-pathogen interactions that could drive clinical manifestations. The preferential replication in RD cells (peak RNA at 12 hpi) and delayed release suggest efficient intracellular propagation, potentially enhancing transmissibility. Together, these findings implicate genetic diversity and modulated host responses as key factors in CVA12’s evolution, underscoring the need to monitor its pathogenic potential in HFMD outbreaks. There were several limitations in the study. Only 9 samples positive for CVA12 determined by rRT-PCR were collected. For the scarce of CVA12 positive specimens, deep insights of CVA12 are limited, especially in terms of evolutionary dynamics, recombinant patterns, and propagation characteristics. Among the 9 samples, four samples with CT values of 29.9–35 failed in library construction of mNGS and in virus isolation because of the low total RNA quantity, which can be explained by low viral load in clinical samples or viral degrration under long storage. Therefore, more data are urgent. Secondly, recombination events (e.g., with CVA5/EV-A71) and host gene responses (e.g., JUN/FOS upregulation) were observed, their functional consequences remain unvalidated. Reverse genetics and mechanistic studies are needed to clarify recombination’s evolutionary role and host-pathogen interactions. Expanded surveillance, specific mechanisms of receptor usage, animal models *in vivo* are essential to fully understand CVA12’s ecology and adaptation in the future.

Overall, this study underscores the complex nature of the CVA12 virome in clinical specimens and its genetic diversity. The findings also emphasize the phylogenetic evolutionary dynamics and recombinant events in unveiling the mechanisms of CVA12 diffusion. Further research is warranted to explore the functional implications of infection and adaptation, as well as to better understand the mechanisms to CVA12 infections.

## Data Availability

The datasets presented in this study can be found in online repositories: GenBank database under the accession numbers PQ726936-PQ726940, while the meta-transcriptome data, which excluded human genomic data, were submitted to the NCBI Sequence Read Archive (SRA) under accession numbers SRR31678006-SRR31678010.

## Footnotes

^1^ https://blast.ncbi.nlm.nih.gov/Blast.cgi
