# Supplementary material for: The genetic and proliferation characterization analysis of novel coxsackievirus A12 in Beijing, China
Source: Front Microbiol. 2025 Oct 8;16:1665461. doi: 10.3389/fmicb.2025.1665461 (PMC12540438; doi:10.3389/fmicb.2025.1665461)

Table S1. Primers and names for amplifying the full-length genomic sequencing of five CVA12 strains.

Primer	Position(nt)	Sequence(5'-3')	Orientation
0001S48		GGGGACAAGTTTGTACAAAAAAGCA<br>GGCTTTAAAACAGCTCTGGGGTT	F
A12-915H	915-935	GCTGTCTCTCTCAATGCATCT	R
A12-864F	864-884	CTAGCAGGCAGGATTTCACCC	F
A12-1765H	1765-1785	ATGGGAGCTGAAACCTCGTCG	R
A12-1694F	1694-1714	CTAAGGCAAGCTGTGCAACAA	F
A12-2647H	2647-2667	CTAGTCTCACTCACTCCATGC	R
A12-2598F	2598-2618	CTAACGCGACTGATGAGAGCA	F
A12-3485H	3485-3505	CCAGTCTGGCATTTGCAACGG	R
A12-3429F	3429-3449	GCTCAAGGGACCTTCTAGTGT	F
A12-4271H	4271-4291	TTCAAGGTCTTCCTGTGAGGC	R
A12-4179F	4179-4199	TACCAGCAGCCAAGGAGAAAG	F
A12-5086H	5086-5106	GCTGGCTTTTCCTCAAGGCTG	R
A12-4959F	4959-4981	CCAAGGTTAGGTATAGTGTGGAC	F

A12-5930H	5930-3952	GGTTTAACCCACTGGATCTCTCC	R
A12-5837F	5837-5856	TCAGTTGGAAAGGTCATCGG	F
A12-6741H	6741-6761	GCGATACACATGGTGTGTGTG	R
A12-6603F	6603-6622	CTCCCTGGCTCACTCTTTGC	F
7500A		GGGGACCACTTTGTACAAGAAAGCTGGG$(T)_{24}$	R

Table S2. The significant 360 up-regulated and 427 down-regulated genes lists, which represent the gene with adjusted p value < 0.05 as well as |log2FoldChange| > 1.

Gene	baseMean	log2FoldChange	lfcSE	stat	pvalue	padj	SYMBOL
ENSG00000003436	428.696279	1.00653313	0.13758988	7.31545915	2.57E-13	1.12E-11	TFPI
ENSG00000006327	516.233508	1.16198843	0.12424063	9.35272451	8.54E-21	7.68E-19	TNFRSF12A
ENSG00000007174	13.7746855	-2.8046238	0.72336653	-3.8771821	0.00010567	0.00078621	DNAH9
ENSG00000007314	655.045308	-1.1503775	0.13424077	-8.5695087	1.04E-17	7.40E-16	SCN4A
ENSG00000007402	109.729808	-1.0057552	0.20593638	-4.8838149	1.04E-06	1.32E-05	CACNA2D2
ENSG00000007944	220.826712	1.43279002	0.15051268	9.51939715	1.74E-21	1.75E-19	MYLIP
ENSG00000010282	10.3781295	-3.4559548	0.91304688	-3.7850792	0.00015366	0.00108176	HHATL
ENSG00000013588	166.347993	1.45105223	0.1874135	7.74251692	9.75E-15	5.16E-13	GPRC5A
ENSG00000015520	11.1825134	2.53189765	0.67706684	3.73950915	0.00018438	0.00126513	NPC1L1
ENSG00000018625	53.04198	-1.097435	0.30348609	-3.6160966	0.00029908	0.00192578	ATP1A2
ENSG00000019549	957.630452	1.60340894	0.08746619	18.3317563	4.62E-75	3.60E-72	SNAI2
ENSG00000023608	49.7912233	1.3599336	0.29969885	4.537667	5.69E-06	6.06E-05	SNAPC1
ENSG00000031691	48.5282093	1.17958943	0.34708987	3.39851302	0.00067753	0.00383998	CENPQ
ENSG00000036448	207.256913	-1.4474933	0.16175726	-8.9485526	3.60E-19	2.81E-17	MYOM2
ENSG00000039987	7.12194981	2.58542165	0.8439251	3.06356767	0.00218715	0.01027827	BEST2
ENSG00000042832	222.045759	-1.0633429	0.15473181	-6.8721675	6.32E-12	2.21E-10	TG
ENSG00000043355	193.16937	1.63844704	0.19165456	8.54895923	1.24E-17	8.80E-16	ZIC2
ENSG00000043591	9.11555406	2.04712102	0.76679213	2.66972096	0.00759143	0.02896423	ADRB1
ENSG00000047346	43.9069861	1.05865845	0.31770477	3.33220827	0.0008616	0.00470558	FAM214A
ENSG00000049540	28.2569785	-1.020205	0.41152264	-2.479098	0.01317151	0.04501078	ELN
ENSG00000049769	112.429079	-1.0046804	0.22931028	-4.381314	1.18E-05	0.00011639	PPP1R3F
ENSG00000054267	253.210874	1.08533937	0.15383117	7.05539301	1.72E-12	6.58E-11	ARID4B
ENSG00000054690	68.9336821	-1.1914374	0.28128942	-4.2356284	2.28E-05	0.00020725	PLEKHH1
ENSG00000057657	13.4884062	4.06321636	0.79441811	5.11470763	3.14E-07	4.59E-06	PRDM1

ENSG00000058866	26.8074083	-1.0646831	0.41766406	-2.5491374	0.01079898	0.03844534	DGKG
ENSG00000059728	214.710135	1.75203178	0.15478248	11.319316	1.05E-29	2.03E-27	MXD1
ENSG00000059804	324.00066	1.56887707	0.15688973	9.99987093	1.53E-23	1.81E-21	SLC2A3
ENSG00000066923	56.4060696	-1.0966513	0.29090903	-3.7697395	0.00016342	0.00113822	STAG3
ENSG00000067082	779.246478	1.52027818	0.09926891	15.3147466	6.09E-53	2.85E-50	KLF6
ENSG00000068028	835.041169	1.0901491	0.1021919	10.6676664	1.44E-26	2.13E-24	RASSF1
ENSG00000069431	95.9550915	-1.0202148	0.25752936	-3.9615474	7.45E-05	0.00058318	ABCC9
ENSG00000070182	18.3487864	-1.783723	0.53303784	-3.3463346	0.00081888	0.00451595	SPTB
ENSG00000070601	1202.43822	-1.0101695	0.08990741	-11.235665	2.72E-29	5.13E-27	FRMPD1
ENSG00000070808	84.7725932	-1.1001261	0.24168487	-4.5519033	5.32E-06	5.71E-05	CAMK2A
ENSG00000071243	145.835802	1.05178031	0.21523478	4.88666525	1.03E-06	1.31E-05	ING3
ENSG00000072182	153.588612	-1.4041945	0.19457259	-7.2168154	5.32E-13	2.19E-11	ASIC4
ENSG00000072952	18.2464567	-1.3322474	0.51678019	-2.5779769	0.00993806	0.03611594	IRAG1
ENSG00000074590	369.942613	1.62709871	0.13426691	12.1183897	8.44E-34	1.94E-31	NUAK1
ENSG00000075891	940.766928	-1.1771934	0.09566502	-12.30537	8.47E-35	1.98E-32	PAX2
ENSG00000076053	157.928215	1.07176713	0.17621885	6.08202311	1.19E-09	2.80E-08	RBM7
ENSG00000077458	83.0917966	1.00075685	0.2396655	4.17564005	2.97E-05	0.00026079	FAM76B
ENSG00000081248	103.49682	-1.3163956	0.23045018	-5.7122787	1.11E-08	2.17E-07	CACNA1S
ENSG00000083817	77.4451086	1.22271454	0.25470918	4.80043379	1.58E-06	1.93E-05	ZNF416
ENSG00000085433	216.506465	1.30250852	0.17958589	7.2528443	4.08E-13	1.72E-11	WDR47
ENSG00000087074	1272.07973	2.16078751	0.07904368	27.3366249	1.56E-164	3.64E-161	PPP1R15A
ENSG00000088827	7.64289377	-3.4193197	1.05791708	-3.2321245	0.00122874	0.00635247	SIGLEC1
ENSG00000089101	19.4162408	-1.3604879	0.51027075	-2.6662078	0.00767123	0.029221	CFAP61
ENSG00000091536	22.5594733	-1.4410342	0.46602065	-3.092211	0.00198672	0.00949724	MYO15A
ENSG00000091622	192.653949	-1.2911663	0.17684932	-7.3009398	2.86E-13	1.22E-11	PITPNM3
ENSG00000092969	124.968797	1.07456698	0.19251629	5.58169383	2.38E-08	4.34E-07	TGFB2

ENSG00000093100	24.928277	-7.699158	1.24789864	-6.1696982	6.84E-10	1.69E-08	NA
ENSG00000095370	60.6123685	-1.245763	0.28740272	-4.3345554	1.46E-05	0.00014083	SH2D3C
ENSG00000095574	265.679987	1.25917985	0.16840233	7.47721137	7.59E-14	3.50E-12	IKZF5
ENSG00000096654	122.783727	1.09722458	0.21268637	5.15888537	2.48E-07	3.70E-06	ZNF184
ENSG00000097096	80.4165255	1.2339535	0.25187908	4.89899155	9.63E-07	1.24E-05	SYDE2
ENSG00000099337	83.7725871	-1.0729269	0.24919322	-4.3056022	1.67E-05	0.00015765	KCNK6
ENSG00000099860	229.881601	2.6077576	0.1742407	14.9664088	1.22E-50	5.53E-48	GADD45B
ENSG00000099994	14.9995885	-1.6110212	0.57777893	-2.7883003	0.00529854	0.02156303	SUSD2
ENSG00000100276	48.4622897	-1.4042155	0.32886114	-4.2699344	1.96E-05	0.00018061	RASL10A
ENSG00000100399	52.7268596	-1.2244199	0.31092577	-3.9379814	8.22E-05	0.00063114	CHADL
ENSG00000100483	98.639047	1.22305826	0.21723563	5.63009977	1.80E-08	3.37E-07	VCPKMT
ENSG00000100628	18.5770888	-2.4752034	0.57702982	-4.2895589	1.79E-05	0.00016793	ASB2
ENSG00000100918	50.9994955	1.07776051	0.29284242	3.6803428	0.00023292	0.0015523	REC8
ENSG00000101180	384.283806	-1.1663605	0.14254431	-8.1824413	2.78E-16	1.71E-14	HRH3
ENSG00000101298	270.928064	-1.4913368	0.15606706	-9.5557436	1.23E-21	1.26E-19	SNPH
ENSG00000101605	52.5927813	-2.6153587	0.35604014	-7.345685	2.05E-13	9.06E-12	MYOM1
ENSG00000101665	249.331149	1.91804119	0.15065528	12.7313242	3.96E-37	1.03E-34	SMAD7
ENSG00000102271	62.0219756	-1.0475168	0.27983776	-3.7433003	0.00018162	0.00125063	KLHL4
ENSG00000102554	232.599052	2.68878307	0.16769692	16.0335862	7.45E-58	3.93E-55	KLF5
ENSG00000102760	22.08034	1.15015273	0.46717322	2.46194064	0.01381875	0.04679052	RGCC
ENSG00000102996	314.082971	-1.2183795	0.14723544	-8.2750423	1.28E-16	8.12E-15	MMP15
ENSG00000104879	242.81811	-1.542815	0.15036541	-10.260438	1.06E-24	1.40E-22	CKM
ENSG00000105605	19.716399	-1.3192716	0.49875773	-2.645115	0.00816631	0.03074201	CACNG7
ENSG00000105650	203.295737	-1.1397553	0.15836668	-7.1969388	6.16E-13	2.51E-11	PDE4C
ENSG00000105708	90.753757	1.62340857	0.25640096	6.33152293	2.43E-10	6.59E-09	ZNF14
ENSG00000105855	13.5084281	1.63021153	0.58176237	2.80219489	0.00507562	0.02077983	ITGB8

ENSG00000105856	236.769542	1.22860982	0.1610976	7.62649366	2.41E-14	1.20E-12	HBP1
ENSG00000105996	29.7041355	1.0244764	0.40480292	2.53080285	0.01138018	0.04004962	HOXA2
ENSG00000106034	55.3188296	1.01784286	0.28563289	3.56346522	0.00036599	0.00228298	CPED1
ENSG00000106366	105.474788	1.3180789	0.21900817	6.01840053	1.76E-09	4.04E-08	SERPINE1
ENSG00000106546	41.1994905	1.33733949	0.41682345	3.20840752	0.00133472	0.00680796	AHR
ENSG00000107742	1081.79273	-1.1472593	0.08696123	-13.192768	9.66E-40	2.82E-37	SPOCK2
ENSG00000107864	85.9050447	1.08077647	0.23110189	4.67662322	2.92E-06	3.34E-05	CPEB3
ENSG00000107984	220.382863	1.24173841	0.14839636	8.36771457	5.87E-17	3.85E-15	DKK1
ENSG00000108018	45.1940926	-1.2760844	0.32700035	-3.9023945	9.52E-05	0.00071809	SORCS1
ENSG00000108387	278.453274	-1.2513478	0.14398822	-8.6906262	3.60E-18	2.65E-16	SEPTIN4
ENSG00000108551	32.9696735	2.29369486	0.40346334	5.68501435	1.31E-08	2.52E-07	RASD1
ENSG00000108691	8.52118613	2.05784448	0.81495442	2.52510376	0.01156641	0.04060898	CCL2
ENSG00000108932	21.8617912	2.13112718	0.4637349	4.59557212	4.32E-06	4.74E-05	SLC16A6
ENSG00000109063	430.52634	-1.3295255	0.12582233	-10.566689	4.25E-26	6.11E-24	MYH3
ENSG00000109705	64.5090144	-1.0015649	0.26828338	-3.7332349	0.00018904	0.001293	NKX3-2
ENSG00000110786	20.8601205	-1.6671009	0.49201081	-3.3883421	0.00070316	0.00396418	PTPN5
ENSG00000111011	671.344343	1.08228584	0.13715958	7.89070548	3.00E-15	1.70E-13	RSRC2
ENSG00000111110	239.507347	-1.0992248	0.16700691	-6.581912	4.64E-11	1.44E-09	PPM1H
ENSG00000111186	80.3779214	-1.2768505	0.24985872	-5.11029	3.22E-07	4.68E-06	WNT5B
ENSG00000111859	82.9747095	1.71121308	0.2699711	6.33850455	2.32E-10	6.32E-09	NEDD9
ENSG00000112139	194.449878	-1.4186072	0.20596667	-6.8875568	5.68E-12	2.00E-10	MDGA1
ENSG00000112218	36.3990485	-1.025698	0.38205863	-2.6846612	0.00726034	0.02797407	GPR63
ENSG00000112245	186.701495	1.52629156	0.28652799	5.32684974	9.99E-08	1.61E-06	PTP4A1
ENSG00000113070	188.359498	1.71265683	0.16642957	10.2905804	7.77E-25	1.05E-22	HBEGF
ENSG00000113240	218.083987	1.10229884	0.1664973	6.620521	3.58E-11	1.13E-09	CLK4
ENSG00000113369	582.358372	5.0105205	0.17352709	28.874572	2.49E-183	8.15E-180	ARRDC3

ENSG00000113389	113.200907	1.11289367	0.20993523	5.30112857	1.15E-07	1.83E-06	NPR3
ENSG00000113916	199.64331	1.38204816	0.18088888	7.64031572	2.17E-14	1.09E-12	BCL6
ENSG00000114019	1270.29413	1.08538246	0.08763289	12.3855603	3.13E-35	7.53E-33	AMOTL2
ENSG00000114315	276.376552	2.12509542	0.16379906	12.9737951	1.72E-38	4.78E-36	HES1
ENSG00000114786	23.9134964	-2.1740359	0.50667491	-4.2907905	1.78E-05	0.00016745	ABHD14A-ACY1
ENSG00000114796	233.410556	1.16884228	0.14721551	7.93966817	2.03E-15	1.16E-13	KLHL24
ENSG00000114853	208.106354	-1.0034829	0.16201269	-6.1938536	5.87E-10	1.48E-08	ZBTB47
ENSG00000115137	45.3874554	1.69916455	0.35880333	4.73564314	2.18E-06	2.58E-05	DNAJC27
ENSG00000115520	99.3177065	1.22018417	0.24063935	5.07059278	3.97E-07	5.60E-06	COQ10B
ENSG00000115596	55.1463492	-1.6382392	0.33487943	-4.8920271	9.98E-07	1.28E-05	WNT6
ENSG00000115738	188.420821	2.26223155	0.16750453	13.5054951	1.45E-41	4.85E-39	ID2
ENSG00000115963	1142.9695	1.70423328	0.10027863	16.9949798	8.95E-65	5.86E-62	RND3
ENSG00000116095	167.261063	1.00960345	0.19190708	5.26089741	1.43E-07	2.24E-06	PLEKHA3
ENSG00000116717	1136.80134	1.03321834	0.09434976	10.9509376	6.58E-28	1.11E-25	GADD45A
ENSG00000116741	279.974271	1.36353243	0.14308976	9.52921065	1.58E-21	1.60E-19	RGS2
ENSG00000116903	267.805425	1.02323077	0.14180478	7.21577074	5.36E-13	2.20E-11	EXOC8
ENSG00000117036	812.906675	1.29347834	0.08671367	14.9166601	2.57E-50	1.14E-47	ETV3
ENSG00000117318	2600.49472	1.35682592	0.07308469	18.5651174	6.16E-77	5.04E-74	ID3
ENSG00000117525	33.4124794	2.35826983	0.43005721	5.48361887	4.17E-08	7.24E-07	F3
ENSG00000118515	127.708458	1.68945839	0.21446915	7.87739579	3.34E-15	1.86E-13	SGK1
ENSG00000118523	1610.51104	2.72840097	0.0976052	27.9534399	5.99E-172	1.63E-168	CCN2
ENSG00000118620	130.996458	1.55541537	0.20772786	7.48775537	7.01E-14	3.27E-12	ZNF430
ENSG00000118985	49.5751715	1.38698516	0.29595219	4.68651765	2.78E-06	3.20E-05	ELL2
ENSG00000119922	92.982754	1.0559799	0.26479514	3.98791276	6.67E-05	0.00053118	IFIT2
ENSG00000119938	151.661051	1.0780954	0.17643841	6.11032157	9.94E-10	2.38E-08	PPP1R3C
ENSG00000120049	19.1470668	-1.3363894	0.50473732	-2.6476928	0.00810431	0.03055076	KCNIP2

ENSG00000120669	53.0546908	-1.7976186	0.37213207	-4.8305932	1.36E-06	1.69E-05	SOHLH2
ENSG00000120738	1803.21136	4.68084911	0.10559774	44.327173	0	0	EGR1
ENSG00000121417	187.681035	1.13685766	0.15605824	7.28482955	3.22E-13	1.37E-11	ZNF211
ENSG00000122367	158.750539	-1.6888479	0.18661671	-9.0498213	1.43E-19	1.17E-17	LDB3
ENSG00000122641	13.2900179	1.99345374	0.59637615	3.34261146	0.00082994	0.00456774	INHBA
ENSG00000122786	440.780798	1.02452176	0.12225156	8.3804389	5.27E-17	3.48E-15	CALD1
ENSG00000122877	34.9976117	2.68955371	0.43295125	6.21213978	5.23E-10	1.33E-08	EGR2
ENSG00000123454	168.569702	-1.6645713	0.17966191	-9.2650203	1.95E-20	1.70E-18	DBH
ENSG00000124171	29.4720821	1.27169285	0.38142275	3.33407704	0.00085583	0.00467876	PARD6B
ENSG00000124257	37.7700727	-1.6758822	0.4186155	-4.0033926	6.24E-05	0.00050149	NEURL2
ENSG00000124302	80.433633	-2.372118	0.30434599	-7.7941489	6.48E-15	3.53E-13	CHST8
ENSG00000124493	43.3798072	-1.4413588	0.35452469	-4.065609	4.79E-05	0.00039543	GRM4
ENSG00000124701	45.2534076	-1.8240342	0.35177135	-5.1852837	2.16E-07	3.26E-06	APOBEC2
ENSG00000124766	1528.56413	1.00919458	0.07318459	13.7897151	2.94E-43	1.02E-40	SOX4
ENSG00000125378	650.403546	1.29579114	0.09891035	13.100663	3.26E-39	9.21E-37	BMP4
ENSG00000125510	132.226332	-1.0900587	0.23195225	-4.6994963	2.61E-06	3.03E-05	OPRL1
ENSG00000125533	9.71343597	-1.9850203	0.7449646	-2.6645835	0.00770837	0.02933064	BHLHE23
ENSG00000125798	40.7180206	1.18223455	0.3659508	3.23058336	0.00123538	0.00637876	FOXA2
ENSG00000125848	135.306753	1.68166504	0.22165572	7.58683365	3.28E-14	1.61E-12	FLRT3
ENSG00000125895	31.8487388	-1.0954428	0.40701976	-2.691375	0.00711582	0.02750849	TMEM74B
ENSG00000125898	235.807868	-1.3083364	0.16089659	-8.1315356	4.24E-16	2.56E-14	FAM110A
ENSG00000125968	1249.70079	1.74314063	0.09626758	18.1072451	2.79E-73	2.08E-70	ID1
ENSG00000126759	13.2776464	1.82928276	0.61995284	2.95068051	0.00317075	0.01403429	CFP
ENSG00000126775	182.205041	1.15811035	0.16821145	6.88484857	5.78E-12	2.04E-10	ATG14
ENSG00000126882	174.606776	-1.0064714	0.20166633	-4.9907756	6.01E-07	8.17E-06	FAM78A
ENSG00000127081	111.70813	1.17585947	0.2448985	4.80141552	1.58E-06	1.93E-05	ZNF484

ENSG00000127084	106.039861	-1.4210596	0.23435735	-6.0636441	1.33E-09	3.13E-08	FGD3
ENSG00000127311	69.7416927	1.29637829	0.26875971	4.82355888	1.41E-06	1.74E-05	HELB
ENSG00000127993	176.7314	1.44527159	0.19984679	7.23189806	4.76E-13	1.99E-11	RBM48
ENSG00000128016	163.214993	1.3685009	0.17992444	7.60597556	2.83E-14	1.41E-12	ZFP36
ENSG00000128342	133.770056	1.72944925	0.20045023	8.6278238	6.25E-18	4.53E-16	LIF
ENSG00000128590	105.920978	1.21474899	0.23179243	5.24067592	1.60E-07	2.48E-06	DNAJB9
ENSG00000128645	30.9331431	1.39874123	0.38484299	3.63457636	0.00027844	0.00180924	HOXD1
ENSG00000128917	16.9659036	-2.078157	0.5759718	-3.6080882	0.00030846	0.00197686	DLL4
ENSG00000128965	51.8571125	-1.2722621	0.3028324	-4.2012084	2.65E-05	0.00023594	CHAC1
ENSG00000129204	151.767837	-1.8715344	0.22105216	-8.4664829	2.53E-17	1.72E-15	USP6
ENSG00000129437	7.43505579	2.464219	0.83850455	2.93882604	0.00329458	0.01449615	KLK14
ENSG00000130182	26.1466554	-1.3529896	0.4403268	-3.0726941	0.00212136	0.01000357	ZSCAN10
ENSG00000130222	233.548548	1.11864772	0.16595725	6.74057746	1.58E-11	5.24E-10	GADD45G
ENSG00000130433	219.186091	-1.0989953	0.17409089	-6.312767	2.74E-10	7.32E-09	CACNG6
ENSG00000131470	345.166294	1.11024919	0.12892991	8.61126169	7.23E-18	5.19E-16	PSMC3IP
ENSG00000131845	223.055199	1.15364415	0.15201021	7.58925422	3.22E-14	1.59E-12	ZNF304
ENSG00000131849	14.7689773	-1.9283991	0.63667281	-3.0288698	0.0024547	0.011302	ZNF132
ENSG00000131931	102.56333	1.38119714	0.2118274	6.52038927	7.01E-11	2.11E-09	THAP1
ENSG00000132010	57.0471679	1.84274601	0.34616864	5.32326104	1.02E-07	1.64E-06	ZNF20
ENSG00000132823	605.137196	1.1784083	0.1063209	11.083506	1.51E-28	2.65E-26	OSER1
ENSG00000133055	152.577914	-2.2200623	0.19870628	-11.172582	5.55E-29	9.99E-27	MYBPH
ENSG00000133731	90.3808615	1.16107217	0.24512954	4.73656573	2.17E-06	2.58E-05	IMPA1
ENSG00000133739	63.4837165	1.15466552	0.33863928	3.40972116	0.00065029	0.0037113	LRRCC1
ENSG00000134531	261.895523	1.51755661	0.14407721	10.5329398	6.09E-26	8.59E-24	EMP1
ENSG00000134780	776.674509	-1.1094311	0.10958287	-10.124129	4.32E-24	5.48E-22	DAGLA
ENSG00000135046	1384.8868	1.45687382	0.08720595	16.7061277	1.18E-62	6.68E-60	ANXA1

ENSG00000135318	29.2150867	1.37736576	0.41987249	3.28043818	0.00103646	0.0055009	NT5E
ENSG00000135482	129.840953	-1.034285	0.2095285	-4.9362498	7.96E-07	1.05E-05	ZC3H10
ENSG00000135744	93.4831018	-1.1468684	0.23000903	-4.9861887	6.16E-07	8.33E-06	AGT
ENSG00000135870	444.714097	1.39395352	0.11952351	11.6625883	1.98E-31	4.21E-29	RC3H1
ENSG00000135999	297.541473	1.00447099	0.14202023	7.07273197	1.52E-12	5.95E-11	EPC2
ENSG00000136244	5.81150836	5.37824432	1.42526152	3.773514	0.00016096	0.00112496	IL6
ENSG00000136367	451.332496	-1.0104944	0.12755024	-7.922325	2.33E-15	1.33E-13	ZFHX2
ENSG00000136603	153.738846	1.11781	0.20756395	5.3853763	7.23E-08	1.20E-06	SKIL
ENSG00000136826	141.528146	1.96635552	0.20970123	9.37693827	6.79E-21	6.29E-19	KLF4
ENSG00000136997	4765.15462	1.44484989	0.07167121	20.1594169	2.22E-90	2.28E-87	MYC
ENSG00000137193	866.765764	1.75303967	0.0940494	18.6395625	1.53E-77	1.32E-74	PIM1
ENSG00000137198	224.294295	-1.2809438	0.16218163	-7.8982051	2.83E-15	1.60E-13	GMPR
ENSG00000137331	289.537628	2.37706396	0.14663869	16.2103466	4.26E-59	2.33E-56	IER3
ENSG00000137474	262.196287	-1.0622798	0.16349344	-6.4973846	8.17E-11	2.44E-09	MYO7A
ENSG00000137801	108.955521	1.5543231	0.25643759	6.06121393	1.35E-09	3.17E-08	THBS1
ENSG00000137955	1244.877	1.10762064	0.10986446	10.0817013	6.66E-24	8.25E-22	RABGGTB
ENSG00000138050	167.442598	1.01988611	0.21580863	4.72588187	2.29E-06	2.70E-05	THUMPD2
ENSG00000138311	9.84972334	1.830082	0.7104583	2.57591755	0.00999745	0.03629952	ZNF365
ENSG00000138316	46.674978	-1.8765557	0.35255215	-5.3227748	1.02E-07	1.64E-06	ADAMTS14
ENSG00000138615	29.2382551	-1.7351412	0.41825736	-4.1485013	3.35E-05	0.00028862	CILP
ENSG00000138650	33.2869027	1.13667393	0.40649671	2.79626842	0.00516964	0.02108566	PCDH10
ENSG00000138764	317.109109	1.06449154	0.15061723	7.06752853	1.58E-12	6.12E-11	CCNG2
ENSG00000139194	22.8749912	-1.330852	0.45603096	-2.918337	0.00351904	0.0153558	RBP5
ENSG00000139263	609.828412	1.10011781	0.09985467	11.0171898	3.16E-28	5.44E-26	LRIG3
ENSG00000139793	111.055768	1.39443442	0.20804962	6.70241269	2.05E-11	6.66E-10	MBNL2
ENSG00000140450	212.84894	1.41719287	0.15967296	8.87559722	6.96E-19	5.30E-17	ARRDC4

ENSG00000141161	279.125538	-1.7264753	0.15887297	-10.867017	1.66E-27	2.68E-25	UNC45B
ENSG00000141404	350.157051	-1.0552128	0.12610512	-8.3677236	5.87E-17	3.85E-15	GNAL
ENSG00000141854	54.782794	-1.237915	0.31462421	-3.9345827	8.33E-05	0.00063894	MISP3
ENSG00000142661	53.1828081	-2.1420522	0.33710331	-6.3542902	2.09E-10	5.76E-09	MYOM3
ENSG00000142765	122.114517	-1.0358722	0.20331707	-5.0948608	3.49E-07	5.02E-06	SYTL1
ENSG00000142867	146.30274	1.2861188	0.21237234	6.05596193	1.40E-09	3.25E-08	BCL10
ENSG00000142871	3249.20615	4.35552165	0.08358346	52.109853	0	0	CCN1
ENSG00000143079	578.647888	1.01435987	0.10820053	9.3748138	6.93E-21	6.37E-19	CTTNBP2NL
ENSG00000143622	547.305206	1.10676656	0.10302162	10.7430513	6.39E-27	9.87E-25	RIT1
ENSG00000143751	351.593191	1.00648327	0.12947194	7.77375596	7.62E-15	4.08E-13	SDE2
ENSG00000143816	70.8647646	-1.3433986	0.31288141	-4.2936351	1.76E-05	0.00016544	WNT9A
ENSG00000143842	834.230644	-1.0386063	0.12418543	-8.3633509	6.10E-17	3.93E-15	SOX13
ENSG00000144120	258.00148	-1.5566066	0.16319982	-9.5380409	1.46E-21	1.48E-19	TMEM177
ENSG00000144596	12.3417505	-1.9248031	0.71965125	-2.6746332	0.0074811	0.02863005	GRIP2
ENSG00000144619	10.9087796	-1.8262217	0.70369772	-2.5951793	0.00945416	0.03473528	CNTN4
ENSG00000144802	367.01199	2.05561343	0.14995706	13.708014	9.09E-43	3.10E-40	NFKBIZ
ENSG00000145242	25.6958068	1.84721183	0.44960032	4.10856429	3.98E-05	0.00033695	EPHA5
ENSG00000145390	124.967749	1.14313535	0.22037024	5.18733991	2.13E-07	3.22E-06	USP53
ENSG00000145632	3548.19297	1.83750151	0.06767352	27.1524467	2.37E-162	4.85E-159	PLK2
ENSG00000145780	205.025104	1.43419958	0.19060233	7.52456476	5.29E-14	2.50E-12	FEM1C
ENSG00000145949	8.09821059	-3.0640177	1.01558307	-3.0170036	0.00255287	0.01169475	MYLK4
ENSG00000146587	440.069228	1.13299033	0.14907504	7.60013425	2.96E-14	1.46E-12	RBAK
ENSG00000146592	441.636824	1.30657162	0.12702732	10.285753	8.17E-25	1.10E-22	CREB5
ENSG00000146674	158.317547	1.05213645	0.18835663	5.58587413	2.33E-08	4.25E-07	IGFBP3
ENSG00000146757	141.698714	1.56949175	0.28047183	5.59589808	2.19E-08	4.03E-07	ZNF92
ENSG00000147234	27.7448732	-1.0374217	0.41256161	-2.5145862	0.01191721	0.0415997	FRMPD3

ENSG00000147378	8.13264773	-2.1179905	0.85280776	-2.4835497	0.01300802	0.04464609	FATE1
ENSG00000148154	982.585376	1.29560534	0.0915096	14.1581364	1.66E-45	6.33E-43	UGCG
ENSG00000148604	101.31103	-1.5539747	0.24912571	-6.237713	4.44E-10	1.15E-08	RGR
ENSG00000148677	496.651261	3.84796442	0.14422236	26.6807762	7.87E-157	1.17E-153	ANKRD1
ENSG00000148926	704.801662	2.80659289	0.10453501	26.8483524	8.82E-159	1.60E-155	ADM
ENSG00000149527	29.2942792	-1.8459708	0.4852805	-3.8039254	0.00014242	0.00101222	PLCH2
ENSG00000149781	86.5318716	1.01047825	0.2495442	4.0492957	5.14E-05	0.00042191	FERMT3
ENSG00000150347	451.658437	1.03786411	0.1190528	8.71767948	2.84E-18	2.09E-16	ARID5B
ENSG00000150457	480.799184	1.29549549	0.11102845	11.66814	1.85E-31	3.99E-29	LATS2
ENSG00000150893	1289.58303	-1.3162083	0.07871116	-16.722003	9.06E-63	5.30E-60	FREM2
ENSG00000150938	1035.80737	1.22348844	0.09617875	12.7209845	4.52E-37	1.16E-34	CRIM1
ENSG00000151006	199.508152	-1.3565317	0.21805112	-6.2211637	4.93E-10	1.26E-08	PRSS53
ENSG00000151025	50.6668438	1.00001897	0.31941929	3.13074069	0.00174366	0.00851947	GPR158
ENSG00000151117	58.3540634	-1.2115752	0.28702731	-4.2211146	2.43E-05	0.00021887	TMEM86A
ENSG00000151239	178.213518	1.08631616	0.23933908	4.5388164	5.66E-06	6.03E-05	TWF1
ENSG00000151332	107.437427	1.09307377	0.23692896	4.61350853	3.96E-06	4.40E-05	MBIP
ENSG00000151657	92.8355033	1.10640003	0.25446585	4.34793128	1.37E-05	0.0001335	KIN
ENSG00000152454	127.755512	1.13830121	0.19886162	5.72408686	1.04E-08	2.05E-07	ZNF256
ENSG00000152475	39.5547238	-1.2014915	0.37318041	-3.2195998	0.0012837	0.00658257	ZNF837
ENSG00000152669	140.616705	-1.3183752	0.21953975	-6.0051775	1.91E-09	4.34E-08	CCNO
ENSG00000152804	27.9221232	1.0360887	0.40887313	2.53401021	0.01127654	0.03974718	HHEX
ENSG00000153234	116.732391	2.65683732	0.24845784	10.6933126	1.09E-26	1.66E-24	NR4A2
ENSG00000153563	24.6731744	-1.6807395	0.45776941	-3.6715854	0.00024105	0.00159544	CD8A
ENSG00000153789	9.66577922	-1.7844521	0.72835231	-2.4499848	0.01428622	0.04807502	CIBAR2
ENSG00000154252	14.5089358	-1.5440622	0.5838329	-2.6446988	0.00817637	0.03075156	GAL3ST2
ENSG00000154640	110.448288	1.2612055	0.22987663	5.48644514	4.10E-08	7.15E-07	BTG3

ENSG00000154734	1090.63114	1.46251218	0.09378041	15.5950712	7.86E-55	3.90E-52	ADAMTS1
ENSG00000155090	681.037513	2.29535684	0.10442596	21.980711	4.41E-107	5.15E-104	KLF10
ENSG00000155792	202.192979	-1.3779165	0.16681037	-8.2603767	1.45E-16	9.14E-15	DEPTOR
ENSG00000156097	9.0914061	-2.0857579	0.76667011	-2.7205415	0.00651751	0.02561926	GPR61
ENSG00000157119	131.343864	-2.3903884	0.25426345	-9.4012271	5.39E-21	5.13E-19	KLHL40
ENSG00000157368	34.8360704	-1.6471121	0.41039574	-4.0134726	5.98E-05	0.00048384	IL34
ENSG00000157978	531.085234	-1.0632552	0.11675997	-9.1063337	8.52E-20	7.12E-18	LDLRAP1
ENSG00000158008	37.6408862	-2.1937546	0.40244029	-5.4511308	5.01E-08	8.57E-07	EXTL1
ENSG00000158220	12.3576392	-2.6313977	0.75556179	-3.4827035	0.00049638	0.00295551	ESYT3
ENSG00000158458	24.6382224	-1.16284	0.46451796	-2.5033261	0.01230321	0.04271934	NRG2
ENSG00000158691	65.9061634	1.43786291	0.30229594	4.75647445	1.97E-06	2.36E-05	ZSCAN12
ENSG00000158859	56.9983788	-1.6733409	0.3676309	-4.5516873	5.32E-06	5.72E-05	ADAMTS4
ENSG00000159761	21.4203125	-1.261486	0.48510036	-2.600464	0.00930978	0.03428176	C16orf86
ENSG00000159882	32.3250015	1.07454172	0.3574475	3.00615253	0.00264576	0.01204613	ZNF230
ENSG00000159885	37.8745723	2.24267298	0.37926203	5.91325472	3.35E-09	7.30E-08	ZNF222
ENSG00000159917	44.3270596	1.3613952	0.37475396	3.63277074	0.00028039	0.00181906	ZNF235
ENSG00000160172	37.0276003	-1.0635673	0.37287675	-2.8523294	0.00434001	0.01821198	FAM86C2P
ENSG00000160221	68.2070993	-1.1471705	0.37740825	-3.039601	0.00236892	0.01098062	GATD3A
ENSG00000160298	356.526936	-1.0478613	0.13935434	-7.5194019	5.50E-14	2.59E-12	C21orf58
ENSG00000160325	770.493817	-1.0152141	0.10883797	-9.3277568	1.08E-20	9.57E-19	CACFD1
ENSG00000160539	229.539553	-1.3253253	0.15685172	-8.4495429	2.92E-17	1.99E-15	PLPP7
ENSG00000160678	25.5035809	-1.1265796	0.43776478	-2.5734815	0.0100681	0.0364913	S100A1
ENSG00000160888	3540.81628	1.55022943	0.08729136	17.7592543	1.46E-70	9.97E-68	IER2
ENSG00000161180	13.7093942	2.5281941	0.67773469	3.73035959	0.00019121	0.00130512	CCDC116
ENSG00000162613	1860.42168	1.28532593	0.09980886	12.8778736	6.00E-38	1.61E-35	FUBP1
ENSG00000162630	40.9845011	1.55334599	0.35482297	4.37780569	1.20E-05	0.00011792	B3GALT2

ENSG00000162772	173.707901	1.06699984	0.16748427	6.3707467	1.88E-10	5.22E-09	ATF3
ENSG00000162775	1170.79002	1.339607	0.08471662	15.8128001	2.54E-56	1.30E-53	RBM15
ENSG00000162849	738.310058	-1.0524361	0.10164949	-10.35358	4.03E-25	5.50E-23	KIF26B
ENSG00000162975	188.315942	-2.0588829	0.1924244	-10.699697	1.02E-26	1.56E-24	KCNF1
ENSG00000163376	16.5570237	1.61594578	0.53622063	3.01358378	0.00258182	0.01180424	KBTBD8
ENSG00000163406	18.1547211	-2.4505801	0.58265541	-4.2058823	2.60E-05	0.00023274	SLC15A2
ENSG00000163545	389.557633	2.05295494	0.14253665	14.4029974	4.95E-47	2.03E-44	NUAK2
ENSG00000163602	150.890933	1.09266956	0.22151684	4.93267034	8.11E-07	1.06E-05	RYBP
ENSG00000163659	499.599383	1.66609664	0.10841394	15.3679188	2.69E-53	1.29E-50	TIPARP
ENSG00000163661	39.1651685	2.55140364	0.38006818	6.71301573	1.91E-11	6.23E-10	PTX3
ENSG00000163734	25.2772613	2.08407479	0.48164509	4.32699272	1.51E-05	0.00014511	CXCL3
ENSG00000163874	187.724189	1.87979538	0.17346031	10.8370345	2.30E-27	3.69E-25	ZC3H12A
ENSG00000163877	369.663468	1.08763289	0.12754493	8.52744887	1.50E-17	1.05E-15	SNIP1
ENSG00000163884	42.0811282	-1.5548516	0.36492432	-4.2607509	2.04E-05	0.00018714	KLF15
ENSG00000164122	415.446101	1.12376264	0.13496271	8.3264675	8.33E-17	5.30E-15	ASB5
ENSG00000164220	43.0728436	1.19735781	0.32288354	3.70832717	0.00020863	0.0014117	F2RL2
ENSG00000164284	165.877478	1.07366027	0.1786527	6.00976226	1.86E-09	4.23E-08	GRPEL2
ENSG00000164303	11.6122932	-2.300027	0.7361018	-3.1246045	0.00178044	0.00867207	ENPP6
ENSG00000164442	1440.75344	2.40346854	0.08974523	26.7810173	5.38E-158	8.80E-155	CITED2
ENSG00000164463	103.863726	2.25128425	0.24032946	9.36749174	7.43E-21	6.72E-19	CREBRF
ENSG00000164603	24.4916957	1.113116	0.41661751	2.67179359	0.0075447	0.02881954	BMT2
ENSG00000164741	253.551771	2.02881596	0.1456881	13.9257496	4.42E-44	1.61E-41	DLC1
ENSG00000164949	201.873871	1.63198218	0.18053057	9.03992129	1.57E-19	1.28E-17	GEM
ENSG00000164951	1190.60912	1.4293768	0.08514838	16.7868946	3.04E-63	1.85E-60	PDP1
ENSG00000165244	435.655826	2.2446326	0.11759515	19.0877993	3.19E-81	3.07E-78	ZNF367
ENSG00000165494	557.62211	1.07678047	0.13539067	7.95313648	1.82E-15	1.05E-13	PCF11

ENSG00000165507	53.5353384	-1.1698208	0.29917414	-3.9101668	9.22E-05	0.0006973	DEPP1
ENSG00000165606	41.0698188	-1.1326259	0.34200171	-3.3117552	0.00092713	0.00499678	DRGX
ENSG00000165816	81.4792823	-1.4405896	0.25555306	-5.6371446	1.73E-08	3.25E-07	VWA2
ENSG00000165828	17.2477149	-1.6603386	0.6071929	-2.7344499	0.00624846	0.02473393	PRAP1
ENSG00000165879	47.6160439	-1.1217849	0.31539673	-3.5567423	0.00037548	0.00233152	FRAT1
ENSG00000166159	92.3761106	-1.2963449	0.26477635	-4.8959996	9.78E-07	1.25E-05	LRTM2
ENSG00000166189	455.920122	-1.3313305	0.12677806	-10.501269	8.52E-26	1.19E-23	HPS6
ENSG00000166225	178.192465	1.26633909	0.1822768	6.94734102	3.72E-12	1.35E-10	FRS2
ENSG00000166257	202.389259	-1.0337609	0.16820293	-6.1459147	7.95E-10	1.93E-08	SCN3B
ENSG00000166317	460.447092	-1.5165523	0.11891343	-12.753415	2.98E-37	7.88E-35	SYNPO2L
ENSG00000166450	82.1684022	1.23544312	0.24291992	5.08580407	3.66E-07	5.22E-06	PRTG
ENSG00000166816	10.7715526	-2.6132384	0.80429032	-3.2491233	0.00115761	0.00604975	LDHD
ENSG00000167037	45.1809174	-1.2045337	0.32778496	-3.6747679	0.00023807	0.00157759	SGSM1
ENSG00000167131	43.5911196	-1.1391467	0.4588179	-2.482786	0.01303594	0.04468528	CCDC103
ENSG00000167550	76.759633	1.19341848	0.25701525	4.64337612	3.43E-06	3.86E-05	RHEBL1
ENSG00000167588	18.8922022	-1.9371069	0.53622568	-3.6124844	0.00030328	0.00195128	GPD1
ENSG00000167614	11.9704127	-1.7004073	0.68572801	-2.479711	0.01314889	0.04497827	TTYH1
ENSG00000167772	27.6453194	-1.3830885	0.44156383	-3.1322505	0.00173472	0.00848744	ANGPTL4
ENSG00000167981	48.4729548	1.3952002	0.33616996	4.15028217	3.32E-05	0.00028682	ZNF597
ENSG00000168135	180.574071	-1.0150716	0.1752963	-5.790605	7.01E-09	1.45E-07	KCNJ4
ENSG00000168298	6.10396194	2.55684408	0.96093582	2.66078549	0.00779586	0.02957188	H1-4
ENSG00000168418	50.7872148	-1.5048914	0.31853654	-4.7243919	2.31E-06	2.72E-05	KCNG4
ENSG00000168497	6.91466722	2.1515776	0.82140932	2.61937323	0.00880915	0.03271245	CAVIN2
ENSG00000168509	58.8744678	-1.9064005	0.31312043	-6.0883938	1.14E-09	2.71E-08	HJV
ENSG00000168564	324.809044	1.70647904	0.13820386	12.3475494	5.02E-35	1.19E-32	CDKN2AIP
ENSG00000168621	54.4294553	1.70001261	0.30000134	5.66668341	1.46E-08	2.78E-07	GDNF

ENSG00000168685	33.5580999	1.58804117	0.39394609	4.03111296	5.55E-05	0.00045206	IL7R
ENSG00000168811	30.8533637	1.3930764	0.4014052	3.47049919	0.00051949	0.00306858	IL12A
ENSG00000169184	561.347228	-1.1368085	0.10176414	-11.171012	5.65E-29	1.01E-26	MN1
ENSG00000169249	94.8440223	1.26976535	0.22528289	5.63631506	1.74E-08	3.26E-07	ZRSR2
ENSG00000169429	27.59468	1.74357768	0.4044067	4.31144611	1.62E-05	0.00015434	CXCL8
ENSG00000169758	10.7843087	-2.1984851	0.76991512	-2.8554902	0.00429704	0.01806784	TMEM266
ENSG00000169896	36.1567427	2.06443247	0.38122249	5.41529554	6.12E-08	1.03E-06	ITGAM
ENSG00000170214	112.97777	-1.7235291	0.21593428	-7.9817298	1.44E-15	8.35E-14	ADRA1B
ENSG00000170323	15.4068031	1.47781511	0.53425457	2.76612532	0.00567267	0.02282262	FABP4
ENSG00000170340	176.49276	1.09944924	0.17172348	6.40243964	1.53E-10	4.33E-09	B3GNT2
ENSG00000170345	418.466353	4.2193223	0.17157842	24.5912177	1.57E-133	1.97E-130	FOS
ENSG00000170381	38.1941948	1.94476145	0.36649311	5.30640659	1.12E-07	1.78E-06	SEMA3E
ENSG00000170458	31.3399908	2.69538222	0.42284838	6.3743468	1.84E-10	5.11E-09	CD14
ENSG00000171161	308.815241	-1.0185797	0.14022181	-7.2640604	3.76E-13	1.59E-11	ZNF672
ENSG00000171295	183.527523	1.1451543	0.20337108	5.63086115	1.79E-08	3.36E-07	ZNF440
ENSG00000171357	27.2811987	-1.2477684	0.43374462	-2.8767353	0.00401813	0.01710054	LURAP1
ENSG00000171408	15.1557997	1.9145345	0.58248466	3.28684107	0.00101318	0.00539659	PDE7B
ENSG00000171450	55.8377177	-1.9321946	0.3394333	-5.6924132	1.25E-08	2.42E-07	CDK5R2
ENSG00000171617	631.776621	1.44323302	0.10196962	14.1535592	1.78E-45	6.60E-43	ENC1
ENSG00000171786	38.2611907	1.03289616	0.36008718	2.86846133	0.00412474	0.01746345	NHLH1
ENSG00000171790	70.2891218	-1.1487555	0.29045614	-3.955005	7.65E-05	0.0005951	SLFNL1
ENSG00000171827	74.3795681	1.00298104	0.2560708	3.9168115	8.97E-05	0.00067994	ZNF570
ENSG00000172059	272.428121	1.17368738	0.14078354	8.33682267	7.63E-17	4.90E-15	KLF11
ENSG00000172123	9.89617428	2.76720208	0.7560978	3.65984675	0.00025237	0.00166281	SLFN12
ENSG00000172748	26.6322954	1.24961906	0.40511831	3.08457805	0.00203841	0.00968778	ZNF596
ENSG00000173110	266.518376	3.41457913	0.19018256	17.9542184	4.45E-72	3.17E-69	HSPA6

ENSG00000173210	7.17173529	-2.4925576	0.91561094	-2.722289	0.00648314	0.02550867	ABLIM3
ENSG00000173269	46.7878205	-1.0606835	0.32929504	-3.2210735	0.00127711	0.00656313	MMRN2
ENSG00000173366	6.58374685	5.56020938	1.60654387	3.46097574	0.00053822	0.0031621	NA
ENSG00000173805	123.423941	-1.402151	0.20979059	-6.6835744	2.33E-11	7.53E-10	HAP1
ENSG00000174010	225.295074	1.12355857	0.17138791	6.55564657	5.54E-11	1.69E-09	KLHL15
ENSG00000174151	490.752866	-1.0254564	0.12024477	-8.5280753	1.49E-17	1.05E-15	CYB561D1
ENSG00000174327	59.1994459	-1.0444882	0.2772324	-3.7675547	0.00016485	0.00114628	SLC16A13
ENSG00000174403	303.135144	-1.766822	0.18450608	-9.5759553	1.01E-21	1.05E-19	NA
ENSG00000174500	44.7190983	-1.3357503	0.35323027	-3.7815284	0.00015587	0.00109449	GCSAM
ENSG00000175073	362.613557	1.12369287	0.13709895	8.19621803	2.48E-16	1.53E-14	VCPIP1
ENSG00000175197	186.782066	1.04188267	0.15709848	6.63203512	3.31E-11	1.05E-09	DDIT3
ENSG00000175264	15.887713	-2.0737553	0.5996294	-3.4583949	0.0005434	0.00318797	CHST1
ENSG00000175463	10.0377276	-2.0711133	0.80651565	-2.5679766	0.01022941	0.03692626	TBC1D10C
ENSG00000175536	20.9405236	-1.657071	0.53247515	-3.1120156	0.00185815	0.00899827	LIPT2
ENSG00000175564	19.4131963	2.0356364	0.49335863	4.12607844	3.69E-05	0.00031535	UCP3
ENSG00000175895	91.8354324	1.530833	0.26553079	5.76518075	8.16E-09	1.65E-07	PLEKHF2
ENSG00000175985	141.892908	-1.4872254	0.19249354	-7.7261055	1.11E-14	5.84E-13	PLEKHD1
ENSG00000176024	53.8598868	1.17935864	0.30573594	3.85744194	0.00011458	0.00084214	ZNF613
ENSG00000176349	20.4634471	-1.6969054	0.53002256	-3.2015719	0.0013668	0.00695207	NA
ENSG00000176381	19.9269952	-1.2684708	0.51425546	-2.466616	0.01363965	0.04625105	PRR18
ENSG00000176410	185.455494	-1.1277909	0.16475186	-6.845391	7.63E-12	2.61E-10	DNAJC30
ENSG00000176697	548.983165	1.02299958	0.10198461	10.0309213	1.11E-23	1.35E-21	BDNF
ENSG00000177352	325.628828	-1.387952	0.14344292	-9.675988	3.81E-22	4.11E-20	CCDC71
ENSG00000177427	213.120967	-1.2179701	0.17756846	-6.8591579	6.93E-12	2.39E-10	MIEF2
ENSG00000177606	3659.51387	3.19641347	0.07237184	44.1665331	0	0	JUN
ENSG00000177854	103.985441	-1.1965703	0.23091637	-5.1818339	2.20E-07	3.31E-06	TMEM187

ENSG00000177875	168.321273	-1.0121226	0.20383838	-4.9653193	6.86E-07	9.19E-06	CCDC184
ENSG00000178229	94.5260668	1.23649672	0.22432278	5.51213185	3.55E-08	6.25E-07	ZNF543
ENSG00000178440	171.733346	1.06748036	0.2122123	5.03024725	4.90E-07	6.82E-06	TIMM23B-AGAP6
ENSG00000178922	21.4197629	-2.5647533	0.77113525	-3.3259448	0.00088119	0.0047966	HYI
ENSG00000178947	29.1892534	-2.1885384	0.47329836	-4.6240144	3.76E-06	4.20E-05	SMIM10L2A
ENSG00000179021	68.9822483	1.00130804	0.27165403	3.68596793	0.00022784	0.00152262	C3orf38
ENSG00000179057	93.3757532	-1.6241515	0.25154765	-6.4566357	1.07E-10	3.11E-09	IGSF22
ENSG00000179071	23.6723267	-1.3475542	0.47795677	-2.8194059	0.00481126	0.01985146	CCDC89
ENSG00000179111	184.27469	-1.0061399	0.16505441	-6.0958076	1.09E-09	2.60E-08	HES7
ENSG00000179119	540.448186	1.11872391	0.11190677	9.99692751	1.57E-23	1.85E-21	SPTY2D1
ENSG00000179314	3733.63448	-1.0271231	0.0769623	-13.345796	1.25E-40	3.87E-38	WSCD1
ENSG00000179348	757.93457	1.05611119	0.10896592	9.69212356	3.26E-22	3.55E-20	GATA2
ENSG00000179388	16.9747477	2.34661742	0.60463343	3.88105799	0.000104	0.00077449	EGR3
ENSG00000179627	195.072861	-1.2043754	0.1884292	-6.3916602	1.64E-10	4.63E-09	ZBTB42
ENSG00000179673	37.6402308	-2.2513096	0.40294051	-5.5872011	2.31E-08	4.22E-07	RPRML
ENSG00000179743	30.6692689	-1.9547101	0.42774105	-4.5698445	4.88E-06	5.31E-05	FLJ37453
ENSG00000179833	642.104147	1.08173017	0.10532541	10.2703627	9.58E-25	1.28E-22	SERTAD2
ENSG00000180425	56.7953898	-1.0718218	0.29792419	-3.5976326	0.00032113	0.00204442	C11orf71
ENSG00000180573	378.813965	1.81292199	0.13577129	13.3527638	1.14E-40	3.59E-38	H2AC6
ENSG00000180747	485.928033	1.24923478	0.14674756	8.51281443	1.70E-17	1.18E-15	SMG1P3
ENSG00000180855	87.0298129	1.20612704	0.24584879	4.90597103	9.30E-07	1.20E-05	ZNF443
ENSG00000180921	70.645665	-1.7935279	0.27863116	-6.4369251	1.22E-10	3.50E-09	FAM83H
ENSG00000181007	134.765603	1.10473413	0.22915002	4.82100829	1.43E-06	1.76E-05	ZFP82
ENSG00000181215	5.75346916	-3.6237205	1.26726549	-2.8594801	0.00424336	0.01786402	C4orf50
ENSG00000181291	58.1187079	-1.3282588	0.3560038	-3.7310242	0.0001907	0.00130276	TMEM132E
ENSG00000181472	464.069178	1.16793832	0.12144275	9.61719266	6.77E-22	7.10E-20	ZBTB2

ENSG00000181625	13.8785898	-2.243002	0.6771264	-3.3125308	0.00092456	0.00498787	SLX1B
ENSG00000181722	173.57207	1.21842421	0.17645293	6.90509484	5.02E-12	1.79E-10	ZBTB20
ENSG00000182040	56.0070544	-1.0938924	0.29742034	-3.6779341	0.00023513	0.00156155	USH1G
ENSG00000182103	11.671746	-1.7796715	0.70952042	-2.5082738	0.01213226	0.04224225	FAM181B
ENSG00000182263	795.026171	1.00079301	0.08887978	11.2600746	2.07E-29	3.93E-27	FIGN
ENSG00000182533	54.9257246	-1.0274521	0.29031118	-3.5391406	0.00040143	0.00246091	CAV3
ENSG00000182600	36.8387257	-1.1442672	0.37142355	-3.0807611	0.00206472	0.00978461	SNORC
ENSG00000182759	477.810141	-1.2909999	0.11966314	-10.788618	3.90E-27	6.13E-25	MAFA
ENSG00000182870	49.0243862	-1.1720634	0.32657579	-3.5889476	0.00033202	0.00210311	GALNT9
ENSG00000183044	379.639295	-1.2486184	0.1403433	-8.8968865	5.74E-19	4.41E-17	ABAT
ENSG00000183114	83.1351094	-1.005184	0.24559098	-4.0929189	4.26E-05	0.00035793	FAM43B
ENSG00000183246	44.4838926	-1.3116332	0.36064899	-3.6368692	0.00027597	0.00179393	RIMBP3C
ENSG00000183307	56.634606	-1.3629691	0.3061941	-4.451324	8.53E-06	8.72E-05	TMEM121B
ENSG00000183631	17.1431439	-1.7547827	0.55661821	-3.1525786	0.00161835	0.0080246	PRR32
ENSG00000184113	30.0431827	-1.6288785	0.42660375	-3.818247	0.0001344	0.00096108	CLDN5
ENSG00000184451	215.042058	-1.0439351	0.16220304	-6.4359773	1.23E-10	3.51E-09	CCR10
ENSG00000184492	16.018052	-1.5030228	0.58419111	-2.5728272	0.01008716	0.03653608	FOXD4L1
ENSG00000184545	341.687311	1.29056777	0.1288532	10.0157992	1.30E-23	1.55E-21	DUSP8
ENSG00000184557	207.795424	1.13471643	0.17772085	6.38482439	1.72E-10	4.82E-09	SOCS3
ENSG00000185022	507.689371	2.52743048	0.13309357	18.9898765	2.07E-80	1.88E-77	MAFF
ENSG00000185028	10.2650221	-2.0680221	0.77163242	-2.6800611	0.00736087	0.02828234	LRRC14B
ENSG00000185105	112.025433	-2.2402572	0.22988857	-9.7449699	1.94E-22	2.14E-20	MYADML2
ENSG00000185176	30.9802419	-1.0132858	0.3953925	-2.5627339	0.01038516	0.03729988	AQP12B
ENSG00000185245	43.3690771	-1.1921848	0.39308978	-3.0328563	0.00242251	0.01117892	GP1BA
ENSG00000185262	914.800358	1.04933447	0.08446287	12.4236183	1.95E-35	4.75E-33	UBALD2
ENSG00000185332	37.4672308	-1.1370859	0.35292866	-3.2218577	0.00127362	0.00654938	TMEM105

ENSG00000185559	28.3629184	-1.2411189	0.44948251	-2.7612172	0.00575864	0.02309663	DLK1
ENSG00000185614	61.7958947	-1.3482619	0.30776459	-4.3808222	1.18E-05	0.00011651	INKA1
ENSG00000185710	127.980657	1.17158811	0.29049227	4.03311286	5.50E-05	0.00044912	SMG1P4
ENSG00000185739	76.7682442	-1.8004349	0.2846916	-6.3241589	2.55E-10	6.85E-09	SRL
ENSG00000185947	115.423893	1.08577828	0.22884592	4.74458216	2.09E-06	2.49E-05	ZNF267
ENSG00000185955	37.7493227	3.00530576	0.5037931	5.96535719	2.44E-09	5.44E-08	C7orf61
ENSG00000186056	30.395275	-1.3265658	0.4447277	-2.9828719	0.00285557	0.0128301	MATN1-AS1
ENSG00000186130	121.736122	1.05051861	0.23479841	4.47413003	7.67E-06	7.94E-05	ZBTB6
ENSG00000186765	25.6617714	-1.5328266	0.49515562	-3.0956461	0.00196385	0.0094044	FSCN2
ENSG00000187122	46.3712985	-1.5263011	0.36731563	-4.155285	3.25E-05	0.00028181	SLIT1
ENSG00000187266	350.836185	-1.1268826	0.13757426	-8.1910859	2.59E-16	1.59E-14	EPOR
ENSG00000187479	104.975681	-1.0849467	0.2135017	-5.0816768	3.74E-07	5.32E-06	C11orf96
ENSG00000187616	82.2968433	-1.8086182	0.25834219	-7.0008627	2.54E-12	9.46E-11	MYMK
ENSG00000187720	559.016496	-1.0277405	0.10211817	-10.064228	7.95E-24	9.78E-22	THSD4
ENSG00000187902	13.4810543	-1.6719985	0.6530644	-2.5602352	0.01046014	0.03752169	SHISA7
ENSG00000187922	20.5468559	-1.8101024	0.50929971	-3.5541006	0.00037927	0.00234972	LCN10
ENSG00000188176	305.245369	-1.3335858	0.14026284	-9.5077626	1.95E-21	1.93E-19	SMTNL2
ENSG00000188283	72.6967431	1.00779191	0.28391855	3.54958104	0.00038584	0.00238231	ZNF383
ENSG00000188295	80.8666165	1.34354666	0.24120259	5.57019989	2.54E-08	4.59E-07	ZNF669
ENSG00000188660	10.6764955	-3.9478577	1.03312466	-3.8212791	0.00013276	0.00095142	LINC00319
ENSG00000188763	185.69147	-1.275748	0.16859171	-7.5670865	3.82E-14	1.86E-12	FZD9
ENSG00000188816	26.2887701	-1.1279588	0.42551967	-2.6507794	0.00803063	0.03029391	HMX2
ENSG00000188833	45.6272572	-1.0636206	0.31713824	-3.3538075	0.00079708	0.00441508	ENTPD8
ENSG00000188868	16.7999883	1.41611813	0.50993156	2.77707486	0.00548505	0.02219515	ZNF563
ENSG00000188981	10.7676659	1.69946258	0.6700922	2.5361623	0.01120748	0.03958228	MSANTD1
ENSG00000189042	80.5740784	1.36804261	0.24936274	5.48615498	4.11E-08	7.15E-07	ZNF567

ENSG00000189164	67.0547064	1.26709279	0.27942856	4.5345859	5.77E-06	6.13E-05	ZNF527
ENSG00000189184	428.025689	1.69598267	0.1287314	13.1745842	1.23E-39	3.53E-37	PCDH18
ENSG00000189334	5.56905036	3.31335583	1.11364799	2.97522723	0.00292772	0.01311104	S100A14
ENSG00000196081	46.5596917	1.37647694	0.33030474	4.1672939	3.08E-05	0.00026922	ZNF724
ENSG00000196110	82.9419909	1.35108744	0.23923541	5.64752272	1.63E-08	3.08E-07	ZNF699
ENSG00000196428	1169.02385	1.14292811	0.10179231	11.2280398	2.97E-29	5.40E-27	TSC22D2
ENSG00000196466	91.4336782	1.59528524	0.23699234	6.73137891	1.68E-11	5.56E-10	ZNF799
ENSG00000196646	102.924669	1.46958138	0.23072161	6.36950039	1.90E-10	5.25E-09	ZNF136
ENSG00000196811	470.764522	-1.1630908	0.11873495	-9.7956902	1.17E-22	1.34E-20	CHRNG
ENSG00000196866	8.86394738	3.17171822	1.02004904	3.10937818	0.00187482	0.00906826	H2AC7
ENSG00000196972	38.6770715	-1.1984283	0.36240415	-3.3068835	0.0009434	0.00507113	SMIM10L2B
ENSG00000196990	34.7713837	-1.0578471	0.37151406	-2.8473946	0.00440787	0.0184435	FAM163B
ENSG00000197044	72.6305384	3.14853727	0.32646126	9.64444367	5.19E-22	5.52E-20	ZNF441
ENSG00000197191	19.9440778	-1.3263867	0.51383887	-2.581328	0.0098421	0.03584132	CYSRT1
ENSG00000197372	83.2367573	1.03700216	0.29029574	3.57222655	0.00035396	0.00222147	ZNF675
ENSG00000197406	39.0067159	-1.1791397	0.3464998	-3.4030026	0.0006665	0.00379055	DIO3
ENSG00000197409	24.5025065	2.17778019	0.47626452	4.57262739	4.82E-06	5.25E-05	H3C4
ENSG00000197579	243.389466	1.02887527	0.17161123	5.99538435	2.03E-09	4.58E-08	TOPORS
ENSG00000197647	78.1576966	2.40151351	0.26083988	9.20684951	3.36E-20	2.88E-18	ZNF433
ENSG00000197653	36.3853207	-1.1122327	0.35363663	-3.1451287	0.00166014	0.00819208	DNAH10
ENSG00000197714	127.442903	1.87512217	0.21281637	8.81098669	1.24E-18	9.31E-17	ZNF460
ENSG00000197753	25.4389866	-1.1181186	0.44764679	-2.4977698	0.01249773	0.04329373	LHFPL5
ENSG00000197808	45.9026249	1.19723208	0.33622036	3.56085537	0.00036965	0.00230272	ZNF461
ENSG00000197816	34.941061	-1.3097069	0.39802107	-3.2905467	0.00099993	0.00533817	CCDC180
ENSG00000197852	1112.25425	-1.2158376	0.08359331	-14.544676	6.31E-48	2.65E-45	INKA2
ENSG00000197857	137.03879	2.05665624	0.20289892	10.1363585	3.81E-24	4.87E-22	ZNF44

ENSG00000197859	196.421302	-1.3313184	0.17657718	-7.5395836	4.71E-14	2.26E-12	ADAMTSL2
ENSG00000197933	81.8434763	1.73450511	0.24111712	7.19362059	6.31E-13	2.56E-11	ZNF823
ENSG00000198093	160.237504	1.2805557	0.17293612	7.40479008	1.31E-13	5.94E-12	ZNF649
ENSG00000198125	19.6238502	-1.5457869	0.51163477	-3.0212702	0.00251717	0.01155384	MB
ENSG00000198160	214.647677	1.02130597	0.16515768	6.18382373	6.26E-10	1.56E-08	MIER1
ENSG00000198203	148.77007	-1.0143544	0.21751316	-4.663416	3.11E-06	3.54E-05	SULT1C2
ENSG00000198300	40.8225718	-1.5356883	0.36296047	-4.2310069	2.33E-05	0.00021073	PEG3
ENSG00000198336	84.1334866	-1.3655172	0.24460531	-5.5825327	2.37E-08	4.33E-07	MYL4
ENSG00000198342	92.4979022	3.16533463	0.27954039	11.3233532	1.01E-29	1.96E-27	ZNF442
ENSG00000198408	1288.90607	1.53531552	0.10769424	14.2562458	4.10E-46	1.60E-43	OGA
ENSG00000198535	10.1942096	-2.079397	0.75457296	-2.7557269	0.00585619	0.02339616	C2CD4A
ENSG00000198711	25.8371586	-1.3793693	0.43905207	-3.1416985	0.00167971	0.0082712	SSBP3-AS1
ENSG00000198732	1592.18659	-1.1905513	0.08271623	-14.393201	5.71E-47	2.28E-44	SMOC1
ENSG00000198788	16.3108448	-1.8816252	0.5817682	-3.2343211	0.00121932	0.00631179	MUC2
ENSG00000198807	118.086214	1.05847917	0.20876173	5.07027396	3.97E-07	5.61E-06	PAX9
ENSG00000198934	71.2897494	-1.0925935	0.28095903	-3.8888	0.00010074	0.00075431	MAGEE1
ENSG00000203685	160.435098	-1.6011301	0.18578703	-8.618094	6.81E-18	4.91E-16	STUM
ENSG00000204060	706.076003	-1.4010224	0.10117911	-13.846954	1.33E-43	4.72E-41	FOXO6
ENSG00000204099	377.553769	-1.5298002	0.17069745	-8.9620563	3.19E-19	2.50E-17	NEU4
ENSG00000204524	123.402926	1.21311102	0.19771319	6.13571121	8.48E-10	2.06E-08	ZNF805
ENSG00000204604	251.721862	1.05518276	0.1423969	7.41015276	1.26E-13	5.72E-12	ZNF468
ENSG00000204624	87.6441325	-1.1841198	0.24493449	-4.8344347	1.34E-06	1.66E-05	DISP3
ENSG00000204634	250.397844	-1.1872291	0.16391951	-7.2427569	4.40E-13	1.84E-11	TBC1D8
ENSG00000204920	61.4568488	1.14108854	0.27697643	4.11980372	3.79E-05	0.0003231	ZNF155
ENSG00000204950	12.3282279	-1.6080298	0.64869862	-2.4788549	0.01318049	0.04502105	LRRC10B
ENSG00000205215	276.277878	-1.3186982	0.16436284	-8.0230919	1.03E-15	6.05E-14	NA

ENSG00000205238	27.2275448	-1.6154736	0.46456667	-3.4773772	0.00050634	0.00300176	SPDYE2
ENSG00000205266	42.0151127	-1.813276	0.3987896	-4.5469491	5.44E-06	5.82E-05	NA
ENSG00000205312	287.539222	-1.2198591	0.1738546	-7.0165474	2.27E-12	8.54E-11	NA
ENSG00000205423	93.9931446	1.46205761	0.2266115	6.45182456	1.11E-10	3.20E-09	CNEP1R1
ENSG00000205795	13.1848993	-1.7439618	0.62700744	-2.7814053	0.00541241	0.02195548	CYS1
ENSG00000205923	58.6191936	1.59031063	0.31432488	5.05944885	4.20E-07	5.90E-06	CEMP1
ENSG00000206262	119.769737	-1.1651497	0.20480034	-5.6891981	1.28E-08	2.46E-07	FOXL2NB
ENSG00000206422	25.7617699	-1.6902969	0.4578896	-3.6914945	0.00022294	0.00149308	LRRC30
ENSG00000210082	58514.2372	1.31603525	0.1120489	11.7451864	7.48E-32	1.65E-29	NA
ENSG00000211459	5732.84412	1.07452612	0.09883119	10.8723385	1.56E-27	2.56E-25	NA
ENSG00000213213	24.480626	-1.451885	0.46362194	-3.1316141	0.00173848	0.00849925	CCDC183
ENSG00000213297	137.461178	2.94942224	0.22060719	13.3695656	9.11E-41	2.92E-38	NA
ENSG00000213626	377.938459	-1.2492186	0.13065751	-9.5610166	1.17E-21	1.20E-19	LBH
ENSG00000213762	282.759634	1.1488872	0.15170454	7.57318933	3.64E-14	1.78E-12	ZNF134
ENSG00000213976	18.0630185	1.45618603	0.50467403	2.88539918	0.00390918	0.01671561	NA
ENSG00000213981	35.6274291	-1.3758227	0.40380375	-3.4071569	0.00065643	0.00374112	NA
ENSG00000214039	29.1751811	-1.857474	0.42260506	-4.3952951	1.11E-05	0.00010994	LINC02418
ENSG00000214140	15.3119464	-1.7595553	0.58473844	-3.0091322	0.00261995	0.01195056	PRCD
ENSG00000214357	520.305673	-1.2155309	0.12450115	-9.7632103	1.62E-22	1.80E-20	NEURL1B
ENSG00000214402	44.8426168	-1.9313262	0.36174539	-5.3389102	9.35E-08	1.51E-06	LCNL1
ENSG00000214456	77.7577544	-1.2268884	0.2535702	-4.8384568	1.31E-06	1.63E-05	PLIN5
ENSG00000214617	317.009266	-1.0978401	0.14574101	-7.5328151	4.97E-14	2.38E-12	SLC6A10P
ENSG00000214650	6.94179414	2.15789593	0.82749208	2.60775418	0.00911384	0.03363592	NA
ENSG00000215695	41.9362838	1.37186375	0.46511721	2.94950116	0.00318287	0.01408356	RSC1A1
ENSG00000215769	87.7194071	-1.081934	0.25919032	-4.174284	2.99E-05	0.00026193	ARHGAP27P1-BPTFP1-KPNA2P3
ENSG00000216895	32.6696588	1.01846952	0.37426164	2.72127677	0.00650303	0.02558077	LOC100506302

ENSG00000221995	53.0360389	-1.6174472	0.30605275	-5.2848643	1.26E-07	1.99E-06	TIAF1
ENSG00000223403	114.814219	-1.1257505	0.20357534	-5.5298964	3.20E-08	5.69E-07	MEG9
ENSG00000223547	483.774192	3.29239332	0.15625738	21.0703221	1.49E-98	1.62E-95	ZNF844
ENSG00000223638	13.7361255	2.1435805	0.66552289	3.22089675	0.0012779	0.00656313	RFPL4A
ENSG00000224239	26.2008131	-1.1130948	0.41791464	-2.6634502	0.00773439	0.02940684	NA
ENSG00000224272	53.1569277	-1.4970007	0.30958607	-4.8354911	1.33E-06	1.66E-05	NA
ENSG00000224287	27.6114348	-1.1775113	0.44753492	-2.6311048	0.00851078	0.03180466	MSL3P1
ENSG00000224660	65.8907458	-1.1044439	0.27240823	-4.0543704	5.03E-05	0.00041389	SH3BP5-AS1
ENSG00000224713	8.63297836	-2.2263302	0.81745434	-2.7234918	0.00645958	0.02543431	NA
ENSG00000224957	48.6137843	-1.0162288	0.34397337	-2.9543822	0.00313296	0.01389265	LINC01266
ENSG00000224975	13.616949	-2.3944839	0.66861768	-3.5812452	0.00034196	0.00215609	INE1
ENSG00000225361	48.356338	-1.3064283	0.31342781	-4.1681954	3.07E-05	0.00026845	PPP1R26-AS1
ENSG00000225756	125.492035	-1.3463308	0.21604026	-6.2318514	4.61E-10	1.19E-08	DBH-AS1
ENSG00000226981	11.4622431	-1.6080369	0.65891604	-2.4404277	0.01466988	0.0491237	NA
ENSG00000227128	151.053276	-1.2257309	0.18530588	-6.6146356	3.72E-11	1.17E-09	NA
ENSG00000227487	25.39264	-1.2059428	0.49422981	-2.4400447	0.01468544	0.04915004	NA
ENSG00000228434	20.7915623	-1.5005771	0.47823964	-3.1377095	0.00170274	0.008372	NA
ENSG00000228492	17.3070203	-2.2179576	0.58029627	-3.8221125	0.00013231	0.00094904	NA
ENSG00000228544	88.5734557	-1.2101858	0.23459818	-5.1585473	2.49E-07	3.71E-06	CCDC183-AS1
ENSG00000228903	26.5939002	-1.5733032	0.45960235	-3.4231835	0.00061892	0.00356332	NA
ENSG00000229089	25.9504691	1.33703864	0.44826428	2.98270173	0.00285716	0.01283371	ANKRD20A8P
ENSG00000229183	19.6304136	-1.6361237	0.63341295	-2.5830285	0.00979372	0.03572625	PGA4
ENSG00000229292	11.9852891	2.52129315	0.65796783	3.83193985	0.00012714	0.00092079	RFPL4AL1
ENSG00000229953	191.683522	-1.228306	0.1630744	-7.5321813	4.99E-14	2.38E-12	NA
ENSG00000230882	304.741349	-1.2372535	0.1370015	-9.0309488	1.70E-19	1.38E-17	NA
ENSG00000231064	31.452485	-1.3115668	0.43180281	-3.0374207	0.00238612	0.01103573	NA

ENSG00000231240	66.6515037	-1.387769	0.27180579	-5.1057375	3.30E-07	4.78E-06	NA
ENSG00000231419	24.2509242	-1.8377623	0.55761412	-3.2957599	0.00098156	0.00525361	LINC00689
ENSG00000231680	46.5096226	-1.9640428	0.38424007	-5.1114992	3.20E-07	4.66E-06	NA
ENSG00000231848	7.95352152	3.87344047	0.99790545	3.88157062	0.00010378	0.00077321	NA
ENSG00000231907	15.4137354	-2.7843295	0.66316675	-4.198536	2.69E-05	0.00023782	NA
ENSG00000232931	125.748713	-1.1163498	0.21933966	-5.089594	3.59E-07	5.13E-06	NA
ENSG00000233117	11.6612719	1.84537302	0.67853166	2.71965646	0.00653498	0.02566943	NA
ENSG00000233705	23.5906041	-1.4754999	0.48695208	-3.0300722	0.00244495	0.01126027	NA
ENSG00000233750	19.530435	-1.3163241	0.52814517	-2.4923528	0.01268999	0.04384838	CICP27
ENSG00000233806	25.5105952	-1.1257094	0.44240955	-2.5444962	0.01094355	0.03886397	LINC01237
ENSG00000234338	25.1977119	-1.4017949	0.44629368	-3.1409695	0.0016839	0.00828932	NA
ENSG00000234678	15.1734028	-1.5364547	0.58037467	-2.6473496	0.00811254	0.0305677	NA
ENSG00000235098	22.6853709	-1.3517977	0.51730762	-2.6131409	0.00897143	0.03317767	ANKRD65
ENSG00000235138	12.0095295	-2.3481442	0.73769088	-3.1831005	0.00145707	0.00734051	LOC100130548
ENSG00000235314	21.5741182	-1.7041679	0.58493784	-2.9134171	0.00357497	0.01554598	NA
ENSG00000235333	17.2813126	-2.0858504	0.60187471	-3.465589	0.00052907	0.00311505	NA
ENSG00000235859	57.6345938	-1.1373886	0.28545908	-3.9844189	6.76E-05	0.00053722	NA
ENSG00000236104	225.441752	-1.0031253	0.15434323	-6.4993151	8.07E-11	2.41E-09	ZBTB22
ENSG00000236383	11.0160258	-1.6826452	0.68173434	-2.4681831	0.01358008	0.04608725	CCDC200
ENSG00000236393	7.41806802	2.45993338	0.83035274	2.96251611	0.00305136	0.0135756	LOC101927476
ENSG00000237037	46.4450902	-1.0624397	0.32378103	-3.2813524	0.00103311	0.00548666	NDUFA6-DT
ENSG00000237296	241.305776	1.40077388	0.15994814	8.75767551	1.99E-18	1.48E-16	SMG1P1
ENSG00000237624	10.782433	-3.5089719	0.9085455	-3.8621862	0.00011238	0.00082967	OXCT2P1
ENSG00000237940	48.1492975	-1.0884957	0.32164066	-3.3841979	0.00071387	0.00401393	LINC01238
ENSG00000239617	9.18226138	2.81303607	0.78534392	3.58191615	0.00034108	0.00215139	RPL32P27
ENSG00000239704	26.2662455	-2.0171679	0.73383997	-2.7487844	0.00598167	0.02379295	CDRT4

ENSG00000240225	112.82982	1.36948854	0.2459136	5.56898244	2.56E-08	4.62E-07	ZNF542P
ENSG00000240668	8.0613404	3.01268487	0.84323035	3.5727899	0.0003532	0.0022184	KRT8P36
ENSG00000241014	10.9007872	-2.6480406	0.85425647	-3.0998192	0.00193639	0.00930831	GPR199P
ENSG00000242252	14.6069875	2.15676646	0.71008396	3.03734007	0.00238676	0.01103573	BGLAP
ENSG00000242599	24.9147654	1.92039281	0.43607129	4.40385063	1.06E-05	0.00010601	CSAG4
ENSG00000242615	59.7123474	3.31351427	0.35362325	9.37018217	7.24E-21	6.58E-19	NA
ENSG00000242686	42.6416921	-1.0822271	0.33693513	-3.2119748	0.00131826	0.00673448	PDE6B-AS1
ENSG00000242852	114.11442	2.93275934	0.25483083	11.5086521	1.19E-30	2.44E-28	ZNF709
ENSG00000243708	39.1587534	-2.2106125	0.50766878	-4.3544386	1.33E-05	0.0001299	PLA2G4B
ENSG00000244560	32.9026512	-1.4007361	0.46704502	-2.9991459	0.00270738	0.01226722	LOC155060
ENSG00000244879	295.01872	-1.0270858	0.2029917	-5.0597429	4.20E-07	5.90E-06	NA
ENSG00000245025	69.4802121	1.13950666	0.27076068	4.20853821	2.57E-05	0.00023052	NA
ENSG00000245532	1843.95474	1.85878849	0.07114378	26.1272103	1.79E-150	2.44E-147	NEAT1
ENSG00000247765	14.5497157	-1.4512859	0.57400431	-2.5283536	0.01145989	0.04028682	NA
ENSG00000248746	55.6388277	-1.625554	0.32353479	-5.0243559	5.05E-07	7.01E-06	ACTN3
ENSG00000249574	25.7013509	-1.0719338	0.43037884	-2.490675	0.01275007	0.04398169	LOC442497
ENSG00000249592	76.4195448	-1.0006506	0.32761635	-3.0543365	0.00225559	0.01055144	LOC100129917
ENSG00000249773	21.5995925	1.10439765	0.44445529	2.48483408	0.01296118	0.04455032	NA
ENSG00000249846	6.19521507	-2.6213354	1.00512877	-2.6079598	0.00910837	0.0336233	LINC02021
ENSG00000249906	11.4663385	-1.7750578	0.68504128	-2.5911691	0.00956505	0.03505613	LOC100288866
ENSG00000250120	20.2661639	-1.314691	0.51433092	-2.556119	0.01058469	0.03784298	PCDHA10
ENSG00000250135	56.5484816	1.03521167	0.33809759	3.06187238	0.00219957	0.01032777	NA
ENSG00000251022	142.88429	1.07704132	0.22611372	4.76327276	1.90E-06	2.28E-05	THAP9-AS1
ENSG00000251364	18.0762112	-1.3900897	0.5309289	-2.6182219	0.00883893	0.03279883	LOC100506258
ENSG00000251562	458.977742	1.74148036	0.15134843	11.5064313	1.22E-30	2.47E-28	MALAT1
ENSG00000251867	26.2172115	1.08602502	0.43377517	2.50365877	0.01229165	0.04269732	NA

ENSG00000253861	118.543119	-1.2245605	0.21115292	-5.7994012	6.66E-09	1.38E-07	NA
ENSG00000253955	20.5758146	-1.809436	0.50391885	-3.5907289	0.00032975	0.0020904	LOC285593
ENSG00000254290	65.4204527	-1.3486745	0.27426314	-4.9174471	8.77E-07	1.14E-05	NA
ENSG00000254389	87.4209286	-1.8589442	0.25301219	-7.3472515	2.02E-13	8.97E-12	RHPN1-AS1
ENSG00000254585	112.282771	-1.4491084	0.22454398	-6.4535615	1.09E-10	3.17E-09	MAGEL2
ENSG00000254806	26.7007781	1.79881149	0.41767371	4.30673862	1.66E-05	0.00015703	SYS1-DBNDD2
ENSG00000254815	26.8751833	-1.3045897	0.45009857	-2.8984534	0.00375008	0.01616148	LMNTD2-AS1
ENSG00000255104	6.65729297	3.24418357	1.19663342	2.71109224	0.0067062	0.02619766	NA
ENSG00000255182	108.403912	-1.0691997	0.23651793	-4.5205861	6.17E-06	6.51E-05	NA
ENSG00000255571	47.6786499	-1.0639248	0.32420949	-3.2815966	0.00103221	0.00548369	MIR9-3HG
ENSG00000255663	30.2376117	1.35027289	0.42094193	3.20774145	0.00133782	0.00681949	NA
ENSG00000256258	5.34943481	-2.844437	1.15913563	-2.4539294	0.01413047	0.04765868	NA
ENSG00000256542	48.9203155	-1.0664183	0.32210811	-3.3107465	0.00093047	0.00500822	NA
ENSG00000256683	66.3034512	1.68272875	0.26464959	6.35832746	2.04E-10	5.63E-09	ZNF350
ENSG00000257390	28.6105725	1.70482224	0.63241528	2.6957322	0.00702341	0.0271964	NA
ENSG00000257591	195.526914	3.63073377	0.2152345	16.8687349	7.64E-64	4.81E-61	ZNF625
ENSG00000258056	61.3859901	-1.1570597	0.29930752	-3.8657891	0.00011073	0.00081937	LOC105369779
ENSG00000258057	13.0084835	-2.001265	0.67704513	-2.9558813	0.00311777	0.01383722	BCDIN3D-AS1
ENSG00000258186	115.418059	1.15273684	0.21952073	5.25115262	1.51E-07	2.35E-06	NA
ENSG00000258498	11.5315306	-1.7633348	0.68376002	-2.5788797	0.00991213	0.0360297	DIO3OS
ENSG00000258727	21.4657554	-1.4159808	0.47424551	-2.9857548	0.00282879	0.01273387	LOC102724814
ENSG00000258881	49.496137	-1.1542834	0.35698643	-3.2334098	0.00122322	0.00632996	NA
ENSG00000259075	62.8070157	1.75933011	0.31167902	5.64468563	1.65E-08	3.12E-07	POC1B-GALNT4
ENSG00000259080	26.8708682	1.58103367	0.54359237	2.90849131	0.00363177	0.01574281	NA
ENSG00000259132	23.1440389	-2.1472577	0.68171046	-3.149809	0.00163377	0.00808391	NA
ENSG00000259399	12.8842201	4.2705938	0.89398585	4.77702617	1.78E-06	2.15E-05	TGIF2-RAB5IF

ENSG00000259820	46.8033502	-1.3645834	0.3449769	-3.9555792	7.63E-05	0.00059424	NA
ENSG00000260005	74.2891858	1.69455341	0.2771513	6.1141818	9.71E-10	2.33E-08	NA
ENSG00000260211	13.6419131	-1.9300474	0.63291124	-3.0494756	0.00229241	0.01069964	NA
ENSG00000260220	67.6089765	-1.7795667	0.31620373	-5.6279117	1.82E-08	3.40E-07	CCDC187
ENSG00000260261	49.2694116	-1.2511832	0.32644249	-3.8327828	0.0001267	0.00091804	NA
ENSG00000260267	59.7990672	-1.4501688	0.29908423	-4.848697	1.24E-06	1.56E-05	NA
ENSG00000260272	14.3822332	2.95326215	0.70222934	4.20555219	2.60E-05	0.00023291	NA
ENSG00000260316	26.6335704	-1.3913801	0.43350573	-3.2096003	0.0013292	0.00678189	NA
ENSG00000260500	6.93705323	-2.4166373	0.98159295	-2.4619547	0.01381821	0.04679052	NA
ENSG00000260633	24.0880954	5.20691916	0.75666215	6.88143199	5.93E-12	2.07E-10	NA
ENSG00000260751	15.2937232	1.72708423	0.60655338	2.84737383	0.00440816	0.0184435	NA
ENSG00000260927	124.056439	-1.5552725	0.59426504	-2.6171362	0.0088671	0.03288098	LOC101927556
ENSG00000261052	36.0836209	-1.067798	0.41843191	-2.5519037	0.01071361	0.03822153	SULT1A3
ENSG00000261150	673.532827	-1.5360622	0.13263087	-11.581484	5.12E-31	1.06E-28	EPPK1
ENSG00000261455	43.6084896	-1.3356218	0.32723694	-4.081513	4.47E-05	0.0003727	LINC01003
ENSG00000261488	59.6035799	-1.33246	0.28237256	-4.7188011	2.37E-06	2.78E-05	TBILA
ENSG00000261504	20.4609562	-1.4010797	0.492913	-2.8424482	0.00447685	0.0186836	LINC01686
ENSG00000261505	25.1243632	-2.3963361	0.70075887	-3.41963	0.00062706	0.00360259	NA
ENSG00000261587	45.948034	-1.1512809	0.34149501	-3.3712965	0.00074815	0.00418087	TMEM249
ENSG00000261717	24.42412	1.43072828	0.45034625	3.17695167	0.00148832	0.00747264	NA
ENSG00000261889	11.0051469	3.10607137	0.74654596	4.16058959	3.17E-05	0.00027651	NA
ENSG00000262179	98.0964631	-1.1594145	0.22826179	-5.079319	3.79E-07	5.37E-06	MYMX
ENSG00000262211	8.37247056	3.95373615	1.028028	3.84594209	0.00012009	0.00087712	NA
ENSG00000262468	38.6351813	-1.0455468	0.37969152	-2.7536745	0.00589304	0.02350895	NA
ENSG00000263624	13.0856048	-1.8739918	0.67473083	-2.7773916	0.00547971	0.02218133	NA
ENSG00000264070	18.5595473	-1.349325	0.51803578	-2.6046946	0.00919562	0.03390716	NA

ENSG00000264577	18.399729	-1.7751819	0.55168729	-3.2177321	0.00129208	0.00662143	NA
ENSG00000264956	58.9355962	-1.2811917	0.29601496	-4.3281316	1.50E-05	0.00014453	NA
ENSG00000265688	122.386215	-1.3433363	0.20740316	-6.4769327	9.36E-11	2.76E-09	MAFG-DT
ENSG00000265763	265.597148	-1.1186744	0.16860898	-6.6347262	3.25E-11	1.04E-09	ZNF488
ENSG00000265972	1406.12034	5.60474709	0.12508893	44.8061012	0	0	TXNIP
ENSG00000266086	42.30557	-1.2803548	0.46173054	-2.7729481	0.0055551	0.02244284	NA
ENSG00000266265	34.8535344	-1.2844963	0.40323348	-3.1854901	0.00144509	0.00728689	KLF14
ENSG00000266962	407.547172	-1.0502061	0.16826031	-6.2415553	4.33E-10	1.12E-08	LOC108783654
ENSG00000267041	309.388046	1.32207562	0.13090375	10.0996004	5.55E-24	6.93E-22	ZNF850
ENSG00000267059	17.0150625	4.41098117	0.80178977	5.50141865	3.77E-08	6.62E-07	NA
ENSG00000267128	7.65682814	-3.412247	1.07006835	-3.1888122	0.00142859	0.00721256	RNF157-AS1
ENSG00000267244	54.4315084	-1.3162343	0.3104578	-4.2396561	2.24E-05	0.00020413	LOC100288123
ENSG00000267270	21.0222057	-1.2246793	0.46744001	-2.6199711	0.00879372	0.03268051	PARD6G-AS1
ENSG00000267287	264.787346	-1.0436632	0.14961855	-6.9754936	3.05E-12	1.12E-10	NA
ENSG00000267385	7.14169533	-2.1627145	0.87383598	-2.4749662	0.01332488	0.04541891	NA
ENSG00000267426	29.7997896	1.64010108	0.4062079	4.03759031	5.40E-05	0.00044174	NA
ENSG00000267500	39.3213555	2.02812235	0.40009515	5.0691	4.00E-07	5.63E-06	NA
ENSG00000267523	27.2756791	-1.2070256	0.41686851	-2.8954587	0.00378605	0.01629076	NA
ENSG00000267680	99.956706	1.0015078	0.22208214	4.50962784	6.49E-06	6.84E-05	ZNF224
ENSG00000267749	12.9354805	-1.7074389	0.62959029	-2.7119842	0.00668818	0.02614571	NA
ENSG00000268083	13.9249515	4.72337335	1.00504119	4.69968134	2.61E-06	3.02E-05	NA
ENSG00000268592	19.6667616	-2.419392	0.6549938	-3.6937633	0.00022096	0.00148163	RAET1E-AS1
ENSG00000268869	27.517787	-1.563199	0.43485073	-3.5947944	0.00032465	0.00206363	NA
ENSG00000268996	36.3274357	-1.3457298	0.37561784	-3.5827101	0.00034005	0.00214568	MAN1B1-DT
ENSG00000269388	11.6265543	-2.3052555	0.72456976	-3.1815508	0.00146489	0.00737349	NA
ENSG00000269399	33.7181689	1.87266184	0.3894841	4.80805721	1.52E-06	1.87E-05	NA

ENSG00000269918	50.0609729	-1.0825019	0.31292584	-3.4592922	0.0005416	0.00317965	NA
ENSG00000270195	22.593282	1.9836923	0.50050753	3.96336153	7.39E-05	0.0005796	NA
ENSG00000270276	43.3597918	1.27060935	0.3759712	3.37953909	0.00072607	0.00407139	H4C15
ENSG00000270956	7.90439255	-2.3428208	0.8906425	-2.630484	0.00852634	0.03185554	NA
ENSG00000271270	128.112134	-1.0491385	0.201821	-5.1983615	2.01E-07	3.06E-06	TMCC1-AS1
ENSG00000271576	35.0437563	-1.2719968	0.38262141	-3.3244266	0.00088601	0.00481319	NA
ENSG00000271646	20.3523571	-1.5363163	0.50393306	-3.0486515	0.00229871	0.0107164	NA
ENSG00000271811	12.4103896	2.2460405	0.69038085	3.25333546	0.00114059	0.00596839	NA
ENSG00000271857	11.0321351	2.14076686	0.68354028	3.13188105	0.0017369	0.00849406	NA
ENSG00000272068	17.542134	-1.4334853	0.54289014	-2.6404703	0.0082791	0.03105233	NA
ENSG00000272405	597.3648	-1.081533	0.11245483	-9.617488	6.75E-22	7.10E-20	NA
ENSG00000272414	65.445473	1.31105329	0.33377419	3.92796492	8.57E-05	0.00065432	FAM47E-STBD1
ENSG00000272661	33.9399477	-1.4371808	0.40448749	-3.5530909	0.00038073	0.00235697	NA
ENSG00000272822	35.9102058	4.16061488	0.57558361	7.22851527	4.88E-13	2.03E-11	NA
ENSG00000272953	11.121524	1.85214975	0.66757859	2.77442954	0.00552986	0.0223654	NA
ENSG00000273084	11.8705386	-2.0047334	0.69495679	-2.8846879	0.00391802	0.01673882	NA
ENSG00000273145	20.2015922	-1.3735451	0.50698588	-2.7092374	0.00674381	0.02631917	NA
ENSG00000273253	19.8544488	-1.8300842	0.51490514	-3.5542162	0.00037911	0.00234972	NA
ENSG00000273274	23.1332771	-1.4296393	0.46365035	-3.0834428	0.0020462	0.00971917	ZBTB8B
ENSG00000273802	84.2810245	2.86737203	0.26979278	10.6280532	2.21E-26	3.22E-24	H2BC8
ENSG00000274471	34.6315916	1.50531121	0.38210043	3.93956953	8.16E-05	0.00062816	HERC2P7
ENSG00000274600	125.594461	-1.6723359	0.23618661	-7.0805702	1.44E-12	5.64E-11	RIMBP3B
ENSG00000274641	11.5625664	2.02374597	0.64317741	3.14648174	0.00165248	0.00815919	H2BC17
ENSG00000275088	39.4834397	-1.2895546	0.36373281	-3.5453349	0.00039211	0.00241647	NA
ENSG00000275221	11.3084173	1.98222274	0.68404362	2.89780164	0.00375788	0.01619084	H2AC15
ENSG00000275620	205.874004	-1.9292368	0.17576824	-10.976026	4.98E-28	8.50E-26	FLJ16779

ENSG00000275713	38.7060435	1.8030916	0.36285708	4.96915089	6.72E-07	9.04E-06	H2BC9
ENSG00000275793	272.983353	-1.6626143	0.2067484	-8.0417273	8.86E-16	5.25E-14	RIMBP3
ENSG00000276045	250.655296	-1.1420515	0.14774505	-7.7298797	1.08E-14	5.68E-13	ORAI1
ENSG00000276966	31.0106789	3.13200878	0.43968968	7.12322562	1.05E-12	4.19E-11	H4C5
ENSG00000277011	12.4237885	-1.8948306	0.72923363	-2.5983863	0.00936631	0.03445117	NA
ENSG00000277128	50.3819137	-1.3892007	0.31972404	-4.3449992	1.39E-05	0.00013505	NA
ENSG00000277287	11.6535838	-1.9550753	0.67903415	-2.8792002	0.00398685	0.01698511	NA
ENSG00000278195	48.2798253	-1.5289673	0.32677384	-4.678977	2.88E-06	3.31E-05	SSTR3
ENSG00000278214	16.7054371	-1.4288324	0.56307828	-2.5375378	0.01116353	0.03948243	LINC02139
ENSG00000278291	16.1197926	-1.752845	0.56008397	-3.129611	0.00175038	0.00854465	NA
ENSG00000278768	74.3518157	-1.0203025	0.25169837	-4.0536714	5.04E-05	0.00041492	NA
ENSG00000279159	31.0998656	-1.3060901	0.50296922	-2.5967595	0.00941078	0.03459143	NA
ENSG00000279233	25.8281605	-1.1867627	0.44947607	-2.6403245	0.00828267	0.03105858	NA
ENSG00000279417	9.25163067	-2.3491599	0.92098502	-2.5507038	0.01075057	0.03832831	NA
ENSG00000279484	15.3022392	-1.5538313	0.61499457	-2.5265773	0.011518	0.04045637	NA
ENSG00000279488	37.2902372	-1.0913249	0.3533676	-3.0883558	0.00201267	0.0096045	NA
ENSG00000279520	46.9818696	1.24314185	0.32287639	3.85020988	0.00011802	0.00086429	NA
ENSG00000279592	39.3360478	1.40379306	0.34317654	4.09058573	4.30E-05	0.00036099	NA
ENSG00000279716	70.4300989	-1.3638066	0.3742561	-3.6440464	0.00026838	0.00175366	NA
ENSG00000279759	49.2314025	-1.3440131	0.31445733	-4.2740716	1.92E-05	0.00017779	NA
ENSG00000279765	6.76373667	2.30013423	0.85516729	2.6896892	0.00715186	0.0276219	NA
ENSG00000279789	19.1960857	-1.7718013	0.52929173	-3.3474947	0.00081546	0.00450013	NA
ENSG00000279905	8.18739415	-3.5065353	1.09000357	-3.2169943	0.00129541	0.0066364	NA
ENSG00000280087	8.88600237	1.8719242	0.70869614	2.64136361	0.0082573	0.03099187	NA
ENSG00000280119	95.9854754	-1.8595916	0.26339454	-7.0600995	1.66E-12	6.38E-11	LOC285097
ENSG00000280160	21.078306	-1.3515301	0.52112091	-2.5935059	0.00950029	0.03484221	NA

ENSG00000280303	12.9319935	-1.5648515	0.62613134	-2.4992385	0.01244605	0.04313978	NA
ENSG00000280332	24.1640753	-1.2494456	0.46455845	-2.6895337	0.00715519	0.02762683	NA
ENSG00000280383	69.8899908	1.05206156	0.27498795	3.825846	0.00013032	0.00093806	NA
ENSG00000280434	26.4462532	-1.8078873	0.46860355	-3.8580316	0.0001143	0.00084049	NA
ENSG00000280435	78.1562412	-1.237552	0.24825837	-4.9849356	6.20E-07	8.38E-06	NA
ENSG00000280537	27.777499	1.63849112	0.46661379	3.51145027	0.00044567	0.00269675	NA
ENSG00000280927	50.9738861	-1.1539801	0.32886692	-3.5089578	0.00044987	0.00271713	CTBP1-AS
ENSG00000281912	16.3512959	-1.55838	0.55710237	-2.7972955	0.00515324	0.02104501	LINC01144
ENSG00000282907	22.9837555	-2.2766857	0.56952622	-3.9975082	6.40E-05	0.00051311	NA
ENSG00000282944	24.7036168	-1.2877255	0.43571947	-2.9554004	0.00312264	0.01385505	NA
ENSG00000282988	53.9671309	3.07779192	0.40748826	7.5530812	4.25E-14	2.06E-12	NA
ENSG00000283486	25.5492297	-2.044294	0.46891908	-4.359588	1.30E-05	0.00012726	FAM95C
ENSG00000283515	153.380262	1.13198965	0.23708861	4.77454262	1.80E-06	2.18E-05	NA
ENSG00000283828	36.3779145	-1.1127038	0.38094596	-2.9208967	0.00349026	0.01525127	LOC102724474
ENSG00000284602	20.1826026	1.61866439	0.47230335	3.42717113	0.0006099	0.00351511	NA
ENSG00000284820	99.3491411	-1.7838791	0.25355283	-7.0355323	1.99E-12	7.52E-11	NA
ENSG00000284981	57.432409	-1.2864823	0.30393545	-4.2327485	2.31E-05	0.00020922	UPK3BL2
ENSG00000285188	35.0809502	-1.2571719	0.40761284	-3.0842305	0.00204079	0.00969629	NA
ENSG00000285508	6.2362783	5.48302472	1.50034181	3.65451704	0.00025767	0.00169105	NA
ENSG00000285565	10.0215459	7.16425402	1.38777256	5.16241221	2.44E-07	3.64E-06	NA
ENSG00000285796	40.2309649	-1.506959	0.35993891	-4.1867078	2.83E-05	0.00024947	NA
ENSG00000285901	152.456253	1.30982114	0.18616436	7.03583173	1.98E-12	7.52E-11	NA
ENSG00000286001	28.3623914	1.41848305	0.55252366	2.56728022	0.01024997	0.03695479	NA
ENSG00000286132	110.890567	2.55642521	0.24062068	10.6242957	2.30E-26	3.33E-24	NA
ENSG00000286156	34.8400789	1.22955377	0.37161899	3.30864088	0.0009375	0.00504272	NA
ENSG00000286431	18.4079025	-1.4362051	0.51498228	-2.7888437	0.00528966	0.0215376	NA

ENSG00000286878	38.3421312	-1.3805159	0.36813795	-3.749996	0.00017684	0.00122285	NA
ENSG00000286989	23.476596	-1.1299365	0.45296988	-2.4945068	0.01261323	0.04362919	NA
ENSG00000287356	38.6086509	-1.0488762	0.34678329	-3.024587	0.00248973	0.01144394	NA
ENSG00000287562	36.425357	-1.202805	0.37648325	-3.1948434	0.00139907	0.00708099	NA
ENSG00000287569	47.9824157	1.12047171	0.30087626	3.72402831	0.00019607	0.00133608	NA
ENSG00000287817	11.5422928	-1.9301689	0.68813726	-2.8049185	0.00503293	0.02063085	NA
ENSG00000287853	9.15392713	2.81067241	0.76381003	3.67980557	0.00023341	0.00155494	NA
ENSG00000288187	22.2049878	-1.4844957	0.48747195	-3.0452945	0.00232453	0.01082442	NA
ENSG00000288658	149.355161	-1.0386786	0.1991428	-5.2157477	1.83E-07	2.80E-06	NA

Figure S1. Light micrographs of cytopathic effects of CVA12 strains (strain s7275) at 6h, 24h and 48h post-infection. Mock, normal cells control; CVA12, infected cell culture with s7275 strain. The 100 $TCID_{50}$ of CVA12 stock were used to infect cell lines.

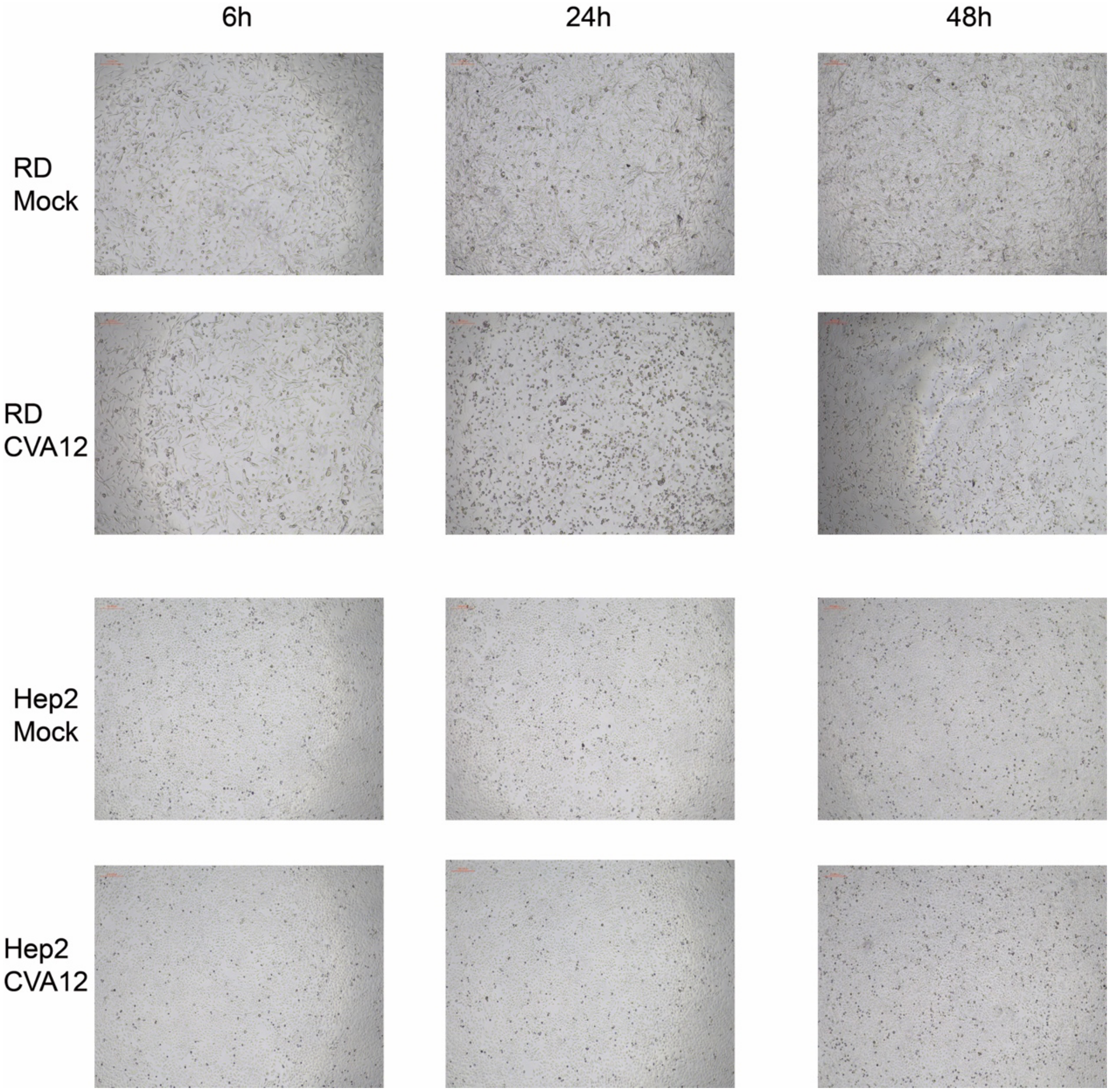

Figure S2. Bayesian phylogenetic trees based on the full-length *VP1* coding region of CVA12. The Bayesian posterior probabilities are indicated at each node. The red colors represent the five CVA12 strains in this study. The distance unit is substitutions per site per year.

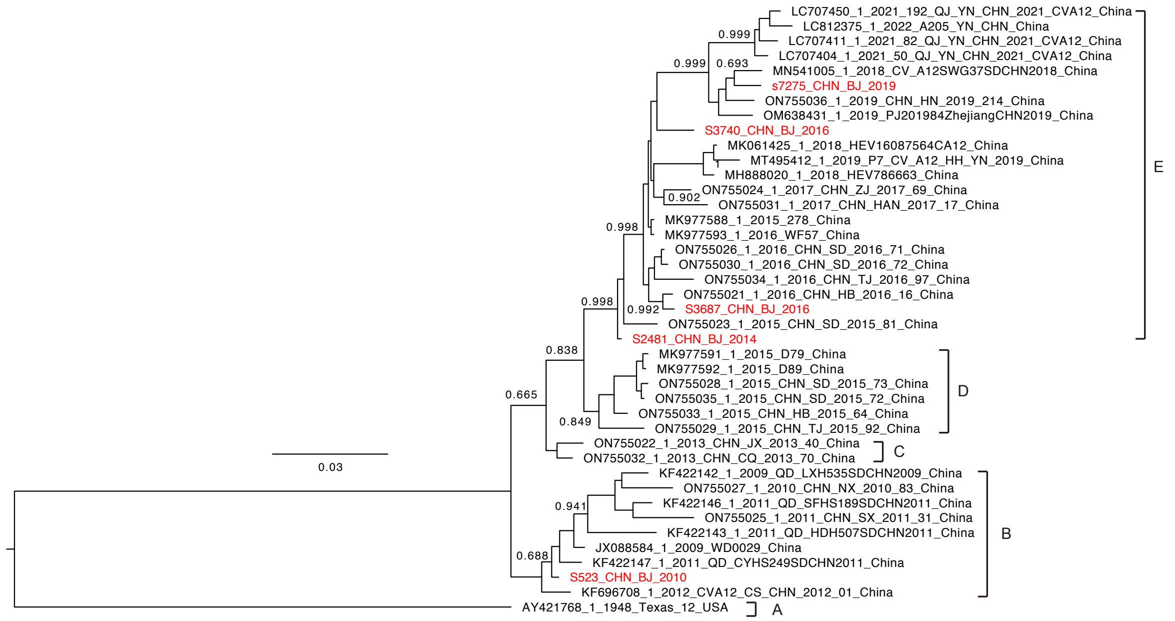

Figure S3. The genes expressed abundantly in RIG-I/MDA5, IFN response, and apoptosis pathways. The representative genes in RIG-I /MDA5 pathway included DDX58, IFIH1, MAVS, TBK1 and IRF3/7, the representative genes of IFN response included IFNB1, MX1, OAS1, STAT1 and ISG15, the representative genes of apoptosis included CASP3, BAX, BCL2, FAS and TP53. Statistical significance was determined by t-test between experiment and control groups. *$P < 0.05$, **$P < 0.01$, ***$P < 0.001$, ****$P < 0.0001$

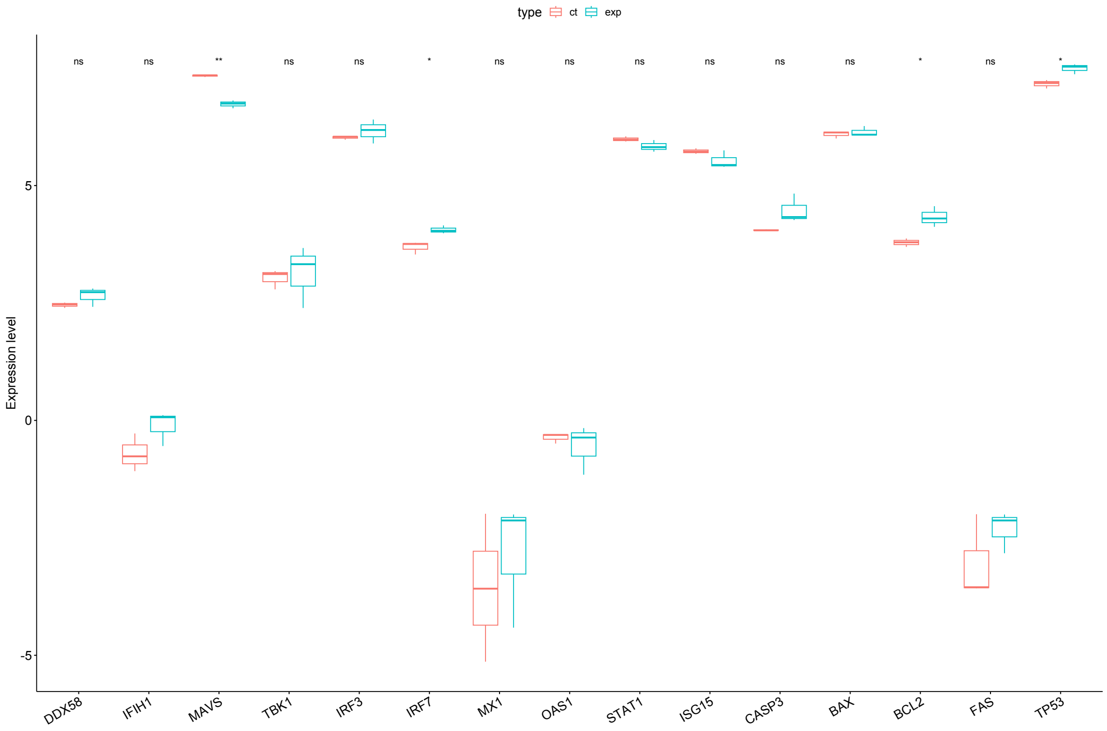

Figure S4. Similarity plot analysis of the strain s7275, other strains (s3687, s3740, s2481 and s523 together) and potential parents. The strain s7275 and others was used as a query sequence, respectively. The strains in the figure is consistent with figure 6.

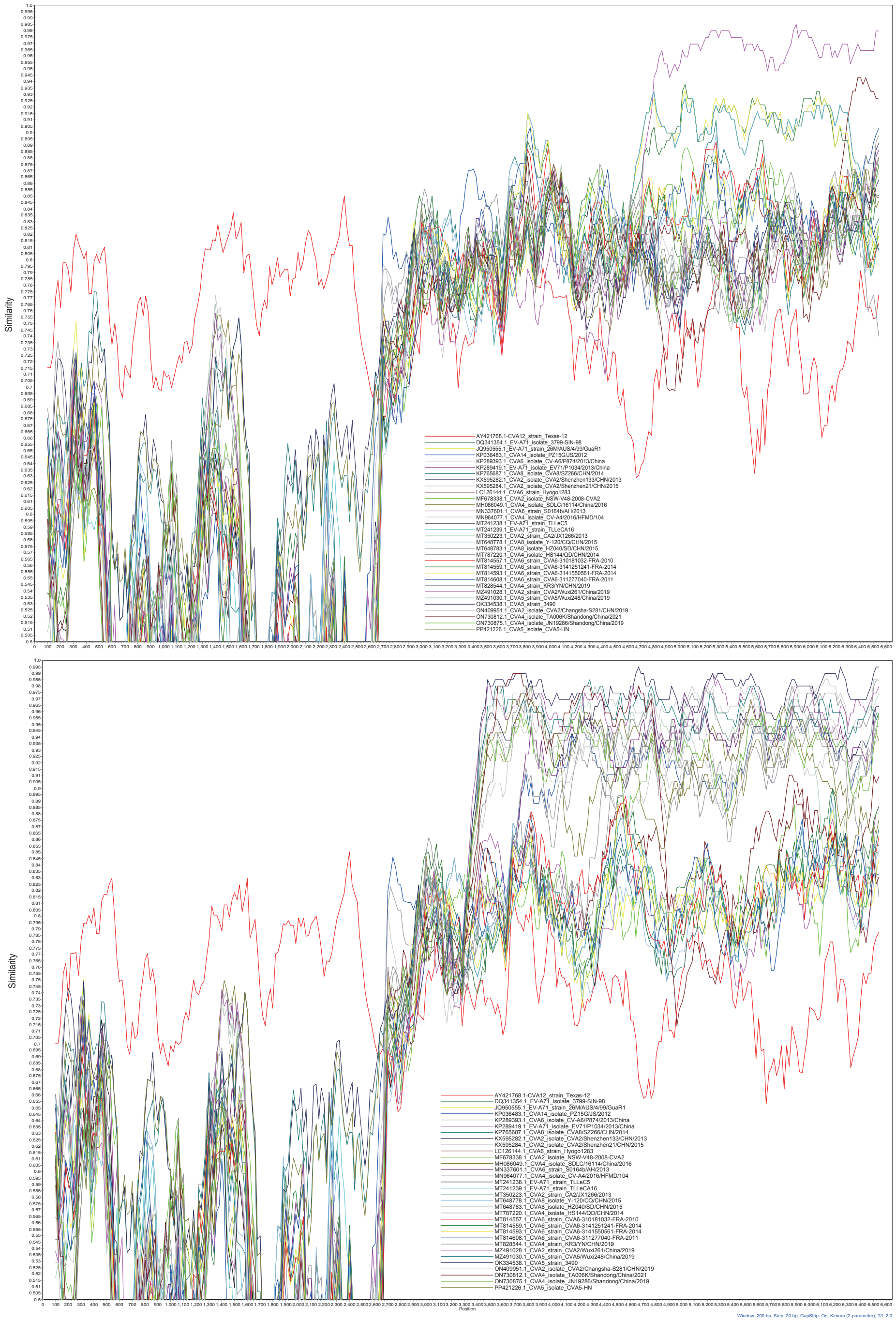

Supplement: Supplementary file 1 [file Data_Sheet_1.PDF]
